# ROP16-mediated activation of STAT6 enhances cyst development of type III *Toxoplasma gondii* in neurons

**DOI:** 10.1371/journal.ppat.1011347

**Published:** 2023-04-17

**Authors:** Joshua A. Kochanowsky, Sambamurthy Chandrasekaran, Jacqueline R. Sanchez, Kaitlin K. Thomas, Anita A. Koshy

**Affiliations:** 1 Department of Immunobiology, University of Arizona, Tucson, Arizona, United States of America; 2 BIO5 Institute, University of Arizona, Tucson, Arizona, United States of America; 3 Postbaccalaureate Research Education Program, University of Arizona, Tucson, Arizona, United States of America; 4 Department of Neurology, University of Arizona, Tucson, Arizona, United States of America; University of New Mexico, UNITED STATES

## Abstract

*Toxoplasma gondii* establishes a long-lived latent infection in the central nervous system (CNS) of its hosts. Reactivation in immunocompromised individuals can lead to life threatening disease. Latent infection is driven by the ability of the parasite to convert from the acute-stage tachyzoite to the latent-stage bradyzoite which resides in long-lived intracellular cysts. While much work has focused on the parasitic factors that drive cyst development, the host factors that influence encystment are not well defined. Here we show that a polymorphic secreted parasite kinase (ROP16), that phosphorylates host cell proteins, mediates efficient encystment of *T*. *gondii* in a stress-induced model of encystment and primary neuronal cell cultures (PNCs) in a strain-specific manner. Using short-hairpin RNA (shRNA) knockdowns in human foreskin fibroblasts (HFFs) and PNCs from transgenic mice, we determined that ROP16’s cyst enhancing abilities are mediated, in part, by phosphorylation—and therefore activation—of the host cell transcription factor STAT6. To test the role of STAT6 *in vivo*, we infected wild-type (WT) and STAT6KO mice, finding that, compared to WT mice, STAT6KO mice have a decrease in CNS cyst burden but not overall parasite burden or dissemination to the CNS. Finally, we found a similar ROP16-dependent encystment defect in human pluripotent stem cell-derived neurons. Together, these findings identify a host cell factor (STAT6) that *T*. *gondii* manipulates in a strain-specific manner to generate a favorable encystment environment.

## Author summary

*Toxoplasma gondii* is a parasite that establishes a latent infection in the brain of its host. In humans who become immunocompromised, this latent infection can reactivate to cause serious neurologic complications. The parasite establishes a latent infection by transitioning from a fast-growing state to a slow growing one. This slow growing form resides within cysts, which are commonly found in neurons. While several parasite factors that drive cyst formation have been identified, only a few host cell genes that promote encystment have been found. Using different models of encystment, including human and mouse primary neuronal cultures, we show that phosphorylation and, thus, activation of the host cell transcription factor STAT6 by the parasite kinase ROP16 is required for efficient encystment of certain *T*. *gondii* strains but not others. Collectively, this work suggests that *T*. *gondii* strain-specific pathways to encystment exist and identifies a direct link between parasite manipulation of the host cell and encystment.

## Introduction

A broad range of microbes—viruses, bacteria, and parasites—establish long-term, latent infections by switching from a rapidly replicating state to a slow-growing quiescent one. While these latent infections enable microbial spread to new hosts, they can also reactivate to cause overt disease when the host becomes immunocompromised. *Toxoplasma gondii*, a common obligate intracellular parasite that infects most warm-blooded animals including humans, switches from a rapidly replicating state, the tachyzoite, to a slow-growing, encysted state, the bradyzoite [1–5]. Bradyzoite-filled cysts are the hallmark of latent infection and are primarily found in neurons in the central nervous system (CNS) and myocytes in skeletal and cardiac muscle [1,3]. For *T*. *gondii*, encystment allows for infection of the definitive host (felids) and passage between intermediate hosts, thereby enabling both the sexual and asexual life cycle of the parasite [1]. In humans, latent infection is generally asymptomatic, but in the setting of acquired immune deficiencies, recrudescence can lead to severe pathology and even death. During the height of the HIV/AIDs epidemic, toxoplasmic encephalitis was the most common focal neurologic finding in AIDS patients [6,7].

Given *T*. *gondii’s* clinical importance, many studies have focused on understanding persistence. These studies have identified many of the parasite factors that define and drive stage conversion and encystment and determined that a range of exogenous stresses (e.g., high pH) can trigger encystment in non-permissive cells (e.g., fibroblasts, macrophages) [8–11]. In permissive cells (neurons and myocytes), high levels of encystment can be achieved without the addition of exogenous stress [12–16]. Collectively, these data suggest that specific host cell pathways promote encystment, but only a single host gene (CDA-1) that influences encystment has been identified in myocytes [15,17]. Glutamine starvation and reactive oxygen species (ROS) production were recently identified as triggers of encystment in murine skeletal muscle cells and IFN-γ stimulated human induced pluripotent stem cells (iPSC)-derived glutamatergic neurons consistent with the observation that encystment may be a generalized response to stress [18,19].

Though many *T*. *gondii* strain types encyst and cause clinically relevant disease [20–24], most work on encystment has been done using type II strains. The ability to compare strains has highlighted that strain-specific effectors influence acute infection [25–29], leading us to question whether strain-specific pathways that influence encystment might also exist. We were particularly interested in the polymorphic effector protein ROP16 for several reasons [30]. ROP16s from strain types I, II, and III translocate into the host cell nucleus and have a functional kinase domain, but only the type I and III alleles (ROP16_I_ and ROP16_III_ respectively), which are 99% identical in their amino acid sequence, cause prolonged phosphorylation and activation of the host transcription factors STAT3, STAT5a, and STAT6 [30–35]. The importance of this STAT activation is strain-specific, as lacking ROP16 does not affect type I strain virulence in mice, but is essential for type III strain survival *in vivo* [31,36,37]. In addition, ROP16_III_-dependent phosphorylation of STAT6 dampens host cell ROS production in human and murine cells, enabling improved type III survival *in vitro* [38]. Considering the profound impact of ROP16 on host cell signaling and tachyzoite survival, we wondered if ROP16 might also influence encystment.

To address this possibility, we compared cyst formation in type II and type III strains that lacked ROP16 (IIΔ*rop16* and IIIΔ*rop16* respectively). We observed that deletion of ROP16 had no effect on type II encystment but significantly decreased type III encystment in both a stress model of encystment and in murine primary neuronal cell cultures (PNCs). Using IIIΔ*rop16* strains complemented with mutated ROP16s, we determined that the ROP16-dependent effect on type III encystment required a ROP16 that is capable of phosphorylating STATs. By using PNCs derived from mice deficient in STAT6 (STAT6KO) [39] and infection of STAT6KO mice with wild-type type III parasites (WT_III_), we identified that efficient encystment required STAT6 *in vitro* and *in vivo*. Finally, we determined that ROP16 was also required for efficient encystment of type III parasites in human neurons. Together these results highlight a mechanism by which an allele of a parasite kinase (ROP16) enhances encystment via activation of a host cell transcription factor (STAT6). To the best of our knowledge, this study identifies the first host cell gene that influences encystment in a strain-specific manner.

## Results

### ROP16 facilitates cyst development in a strain-specific manner

To assess how ROP16 affected encystment, we infected human foreskin fibroblasts (HFFs) with WT_III_ (CEP) [40], IIIΔ*rop16* (CEPΔ*rop16*), or strains where we re-introduced the type III allele of *rop16* (ROP16_III_) or the type II allele of *rop16* (ROP16_II_) [36–38]. Of note, these strains all constitutively express an RFP (WT_III_ expresses mCherry and the others express tdTomato). We induced encystment by placing the infected cultures under alkaline stress (pH 8.2) in combination with CO_2_ deprivation (<5%) and tracked differentiation using immunofluorescent staining of *T*. *gondii* stage-specific surface antigens [41,42] and *Dolichos biflorus* agglutinin (DBA), which binds sugar moieties present on the proteins that form the cyst wall [43]. At 6 days post infection (dpi) the WT_III_ strain no longer expressed the tachyzoite-specific antigen SAG1 [41], instead expressing the bradyzoite-specific surface antigen SRS9 [42] and staining positive for DBA, indicating formation of the cyst wall (**S1 Fig**). In contrast, IIIΔ*rop16* parasites displayed a defect in SRS9 expression and DBA staining, which could be rescued by complementation with ROP16_III_ but not by complementation with ROP16_II_ (**S1 Fig**).

**Fig 1:**
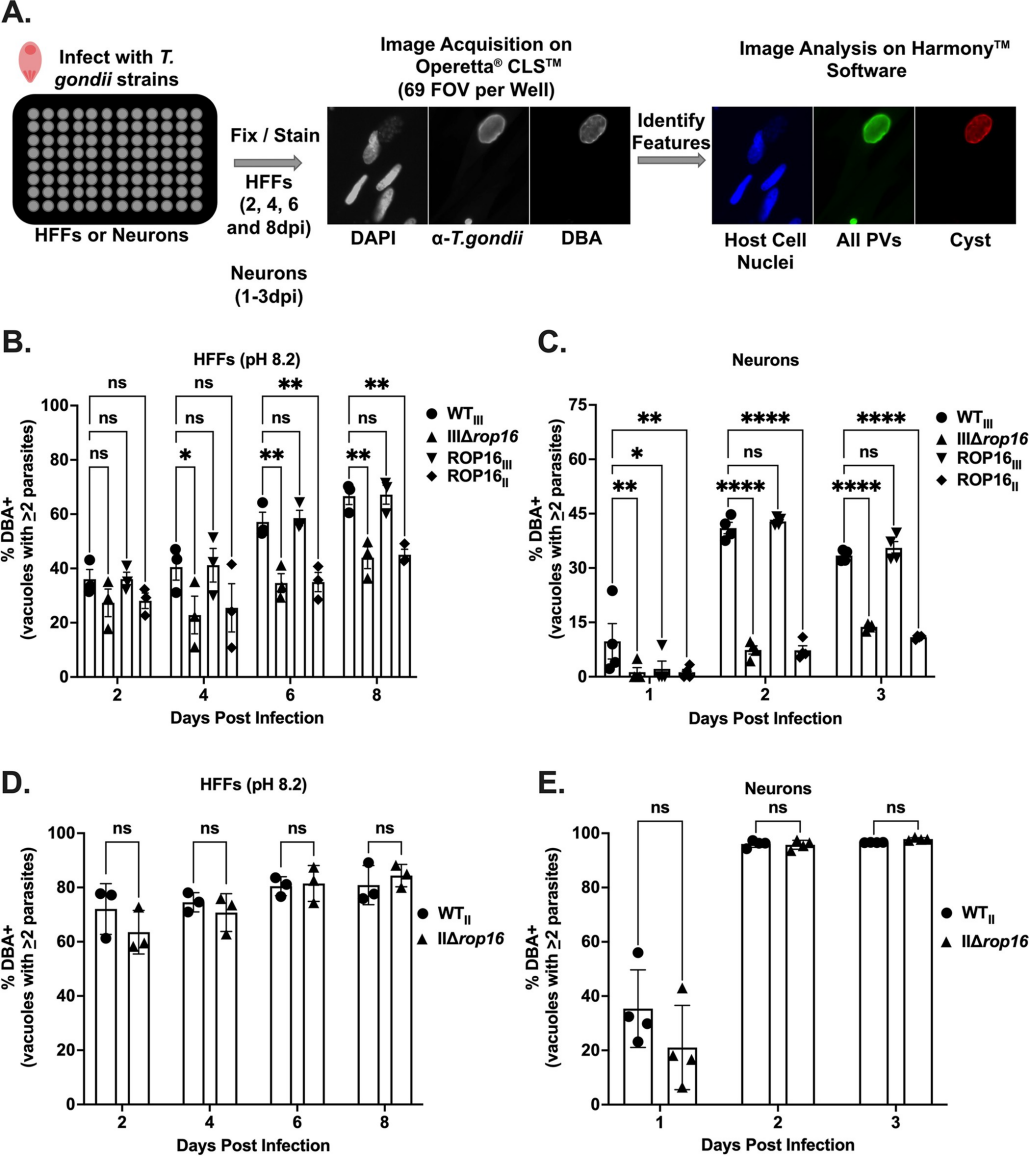
ROP16_III_ facilitates cyst formation of a type III parasites in multiple models of cyst development. (A) Schematic of automated cyst quantification using Operetta CLS and Harmony software. Parasitophorous vacuoles (PVs) were identified by staining with anti-*T*. *gondii* antibody. Cysts were identified by DBA staining. (B) Quantification of encystment of over time in a stress model of encystment for listed type III strains. (C) Quantification of encystment over time as in (B) except in primary neuronal cultures (PNCs). (D) Quantification of encystment over time in stress model of encystment for listed type II parasites. (E) Quantification of encystment over time as in (D) except in PNCs. (B-E) % Cyst = (# of DBA^+^ Vacuoles with ≥2 parasites /# of Total Vacuoles with ≥2 parasites)*100. Bars, mean ± SEM. Black dots = Average % cyst for 1 experiment. (B, D) N = 10 wells/experiment, 3 experiments total. (C, E) N = 5 wells/experiment, 4 experiments total. (B-E) *p≤0.05, **p≤0.005, and ****p≤0.0001. ns = not significant, two-way ANOVA, Dunnett’s multiple comparisons test compared to WT_III_ or WT_II_.

To further characterize and quantify this defect in cyst development, we established an automated imaging pipeline using the Operetta CLS platform and Harmony software (**Fig 1A**). This automated system allowed us to track all parasitophorous vacuoles (PVs) through staining with a polyclonal anti-*T*. *gondii* antibody [44] and cysts through staining with DBA. Using this system, we tracked the encystment rate of our strains over the course of eight days. Compared to WT_III_ and ROP16_III_ parasites, IIIΔ*rop16* and ROP16_II_ parasites showed a significant reduction (30–50%) in encystment at 4–8 dpi (**Fig 1B**). To ensure that this decrease in encystment was not due to a decrease in overall parasite survival of the IIIΔ*rop16* and ROP16_II_ parasites, we used this automated system to track the accumulation of the total number of PVs over time. There were no significant differences in the accumulation of PVs between any of our strains (**S2A Fig**). In addition, the rate of encystment did not vary by the number of parasites within a PV (**S2B Fig**). Collectively, these data indicate that the defect in encystment that we observed is unlikely to be explained by a defect in parasite survival or a delay in stage conversion in the strains that lack ROP16_III_.

Although the alkaline and CO_2_ stress model is commonly used to increase encystment, it is a highly artificial setting. As neurons are the major host cell type for persistent infection in the CNS [45–50], we decided to use PNCs, which prior studies suggest induce encystment without the need for exogenous stress [12,13,16]. To test how ROP16 influenced type III encystment in neurons, we infected PNCs with WT_III_, IIIΔ*rop16*, or the two complemented strains and tracked cyst wall formation using DBA staining. Consistent with what we found in alkaline-stressed HFFs, we observed a defect in encystment in PNCs infected with IIIΔ*rop16* and ROP16_II_ compared to WT_III_ and ROP16_III_-infected cultures (**S3 Fig**). To quantify this defect more robustly, we used our automated cyst quantification system to track the encystment rate over 3 days. Compared to WT_III_ and ROP16_III_ parasites, IIIΔ*rop16* and ROP16_II_ parasites showed a 50–75% reduction in encystment from 1–3 dpi in PNCs (**Fig 1C**). Akin to our findings in the stress model of encystment, in PNCs there were no significant differences in the accumulation of PVs between any of the strains nor did the rate of encystment vary with the number of parasites within a PV (**S2C and S2D Fig**).

To determine if this ROP16-dependent defect in encystment was restricted to the type III strain or was also relevant to genetically distinct *T*. *gondii* strain types, we generated a ROP16 deficient type II strain (PrugniaudΔ*rop16* or IIΔ*rop16* for simplicity) using a CRISPR/CAS9 approach [36,38]. We then tested encystment of WT_II_ (Prugniaud/PRU) [51] and IIΔ*rop16* parasites in the alkaline stress model and PNCs. Unlike the defect observed in the IIIΔ*rop16* strain, deletion of ROP16 in a type II background had no effect on cyst development in stressed HFFs or in PNCs (**Figs 1D, 1E and S4**). Together these results indicate that ROP16 is needed for maximal encystment of type III parasites, but not for type II parasites, suggesting that strain-specific differences in encystment exist.

### ROP16 facilitates encystment through host cell manipulations

**Fig 2:**
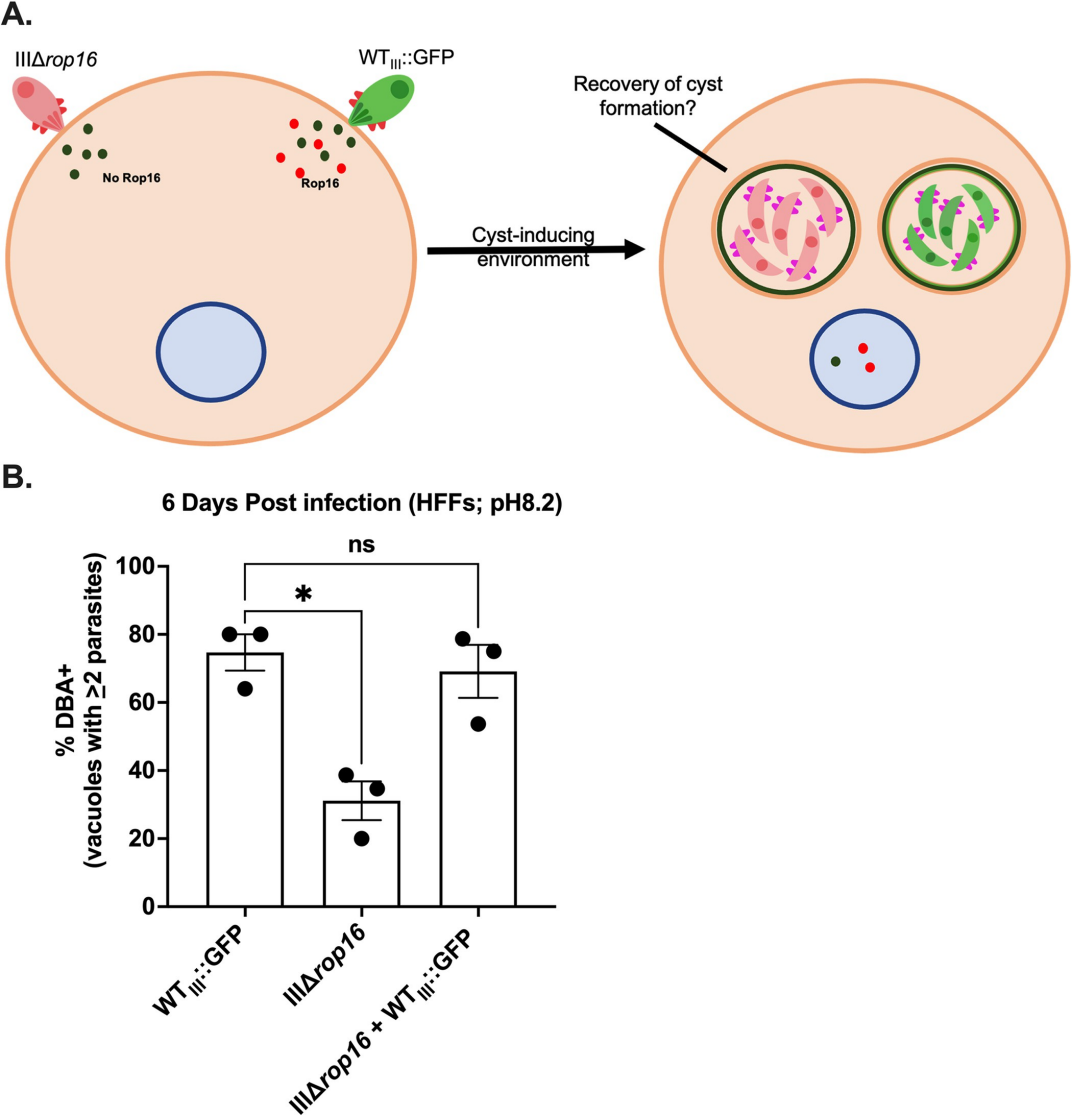
Co-infection of IIIΔ*rop16* with WT parasites restores encystment. (A) Schematic of co-infection experiment. WT_III_:GFP parasites are type III (CEP) parasites that express GFP. The dark circles represent non-ROP16 rhoptry protein secretion. The red circles represent ROP16 secretion by the WT_III_::GFP parasites. (B) Quantification of encystment at 6 dpi in alkaline stress model of encystment. Bars, mean ± SEM. Black dots = Average % cyst for 1 experiment. N = 3 replicates/experiment, 3 experiments total. *p≤0.05, ns = not significant, one-way ANOVA, Dunnett’s multiple comparisons test compared to WT_III_.

While ROP16 is thought to primarily phosphorylate host cell proteins [30], we sought to ensure that our cyst defect was not due to a direct effect of ROP16 on parasite proteins. To accomplish this goal, we tested whether encystment of the IIIΔ*rop16* strain could be restored by co-infection of a host cell with WT_III_ GFP-expressing parasites (WT_III_::GFP) (**Fig 2A**). We hypothesized that if ROP16 facilitates cyst development through host cell manipulations then the IIIΔ*rop16* cyst defect should be rescued in host cells which also house WT_III_::GFP parasites that have injected a functional copy of ROP16. However, if ROP16 is required for phosphorylation of a parasite protein involved in encystment then co-infection with WT_III_::GFP parasites should be insufficient to restore encystment. Co-infection with WT_III_::GFP was sufficient to restore encystment of IIIΔ*rop16* in an alkaline stress model of encystment, indicating that ROP16 facilitates cyst development through host cell manipulations (**Fig 2B**). Together these results indicate that ROP16 enhances encystment via host cell manipulations.

### Efficient encystment requires a ROP16 with an active kinase domain and a leucine at position 503

**Fig 3:**
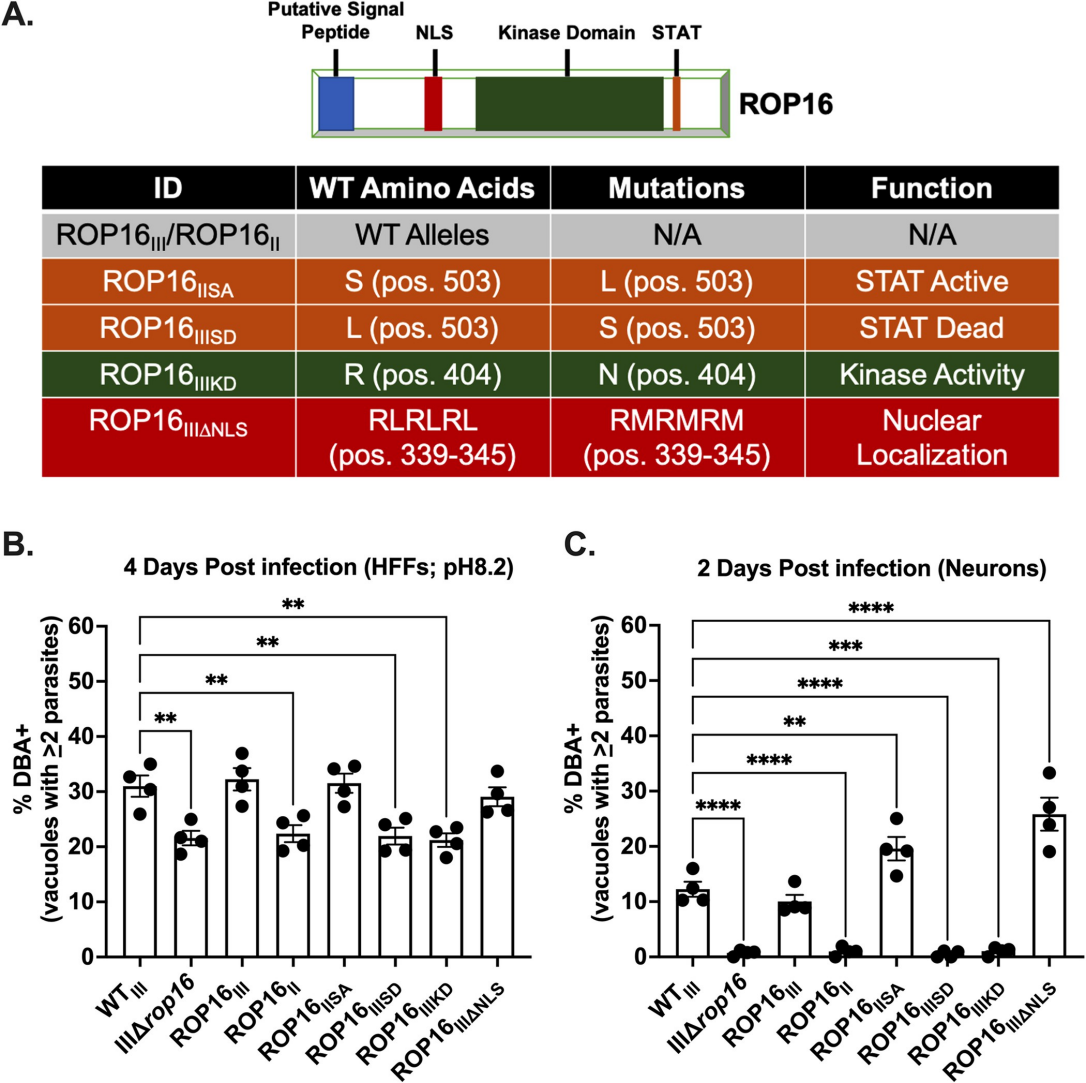
Efficient encystment requires a ROP16 with a functional kinase domain and a leucine at position 503. (A) Schematic of ROP16 and mutations in ROP16 constructs. (B) Quantification of encystment at 4 dpi in alkaline stress model of encystment. Bars, mean ± SEM. Black dots = Average % cyst for 1 experiment. N = 10 wells/experiment, 4 experiments total. (C) Quantification of encystment at 2 dpi in PNCs. Bars, mean ± SEM. Black dots = Average % cyst for 1 experiment. N = 3 wells/experiment, 4 experiments total. (B, C) **p≤0.005, ***p≤0.0005, and ****p≤0.0001, one-way ANOVA, Dunnett’s multiple comparisons test compared to WT_III_.

Having determined that ROP16_III_-dependent host cell manipulation is essential for efficient type III encystment, we next sought to determine what domains or functions of ROP16 were required for this phenotype. To accomplish this goal, we used a previously generated panel of IIIΔ*rop16* complemented strains [38]. This panel of strains includes ROP16 mutants which are catalytically inactive (ROP16_IIIKD_) or lack a nuclear localization sequence (ROP16_IIIΔNLS_) (**Fig 3A**) [38]. In addition, as prior work demonstrated that a single polymorphic amino acid on ROP16 determined the strain-specific activation of STAT3/5a/6 [34], this panel also includes strains which express a ROP16_III_ in which the leucine at position 503 was switched to a serine, rendering it “STAT-dead” (ROP16_IIISD_), and a strain that expresses a ROP16_II_ in which the serine at position 503 was changed to a leucine, rendering it “STAT-active” (ROP16_IISA_) [38] (**Fig 3A**). We tested the ability of this panel of parasites to encyst in the stress model of encystment and in PNCs. In both models, IIIΔ*rop16*, ROP16_II_, ROP16_IIISD,_ and ROP16_IIIKD_ parasites were defective in forming cysts compared to WT_III_, ROP16_III_, or ROP16_IISA_ parasites (**Fig 3B and 3C**). Together these data indicate that efficient *in vitro* encystment is dependent on kinase activity and a leucine at position 503, while the nuclear localization sequence (NLS) is dispensable. Additionally, the ability of ROP16_IISA_ to restore these defects suggested a role for phosphorylation/activation of STATs in cyst development.

### STAT6 is required for efficient encystment *in vitro*

To test the role of individual STATs in type III encystment, we used previously generated STAT3, 5a, and 6 Lentiviral-mediated knock-down HFFs [38] in a cyst assay. Compared to encystment in non-targeting shRNA control HFFs, knock-down of STAT6 significantly reduced encystment of WT_III_ parasites at both 2 and 4 dpi, while knockdown of STAT5a reduced encystment only at 4 dpi (**S5 Fig**). Knock-down of STAT3 had no effect on encystment at 2 or 4 dpi (**S5 Fig**).

**Fig 4:**
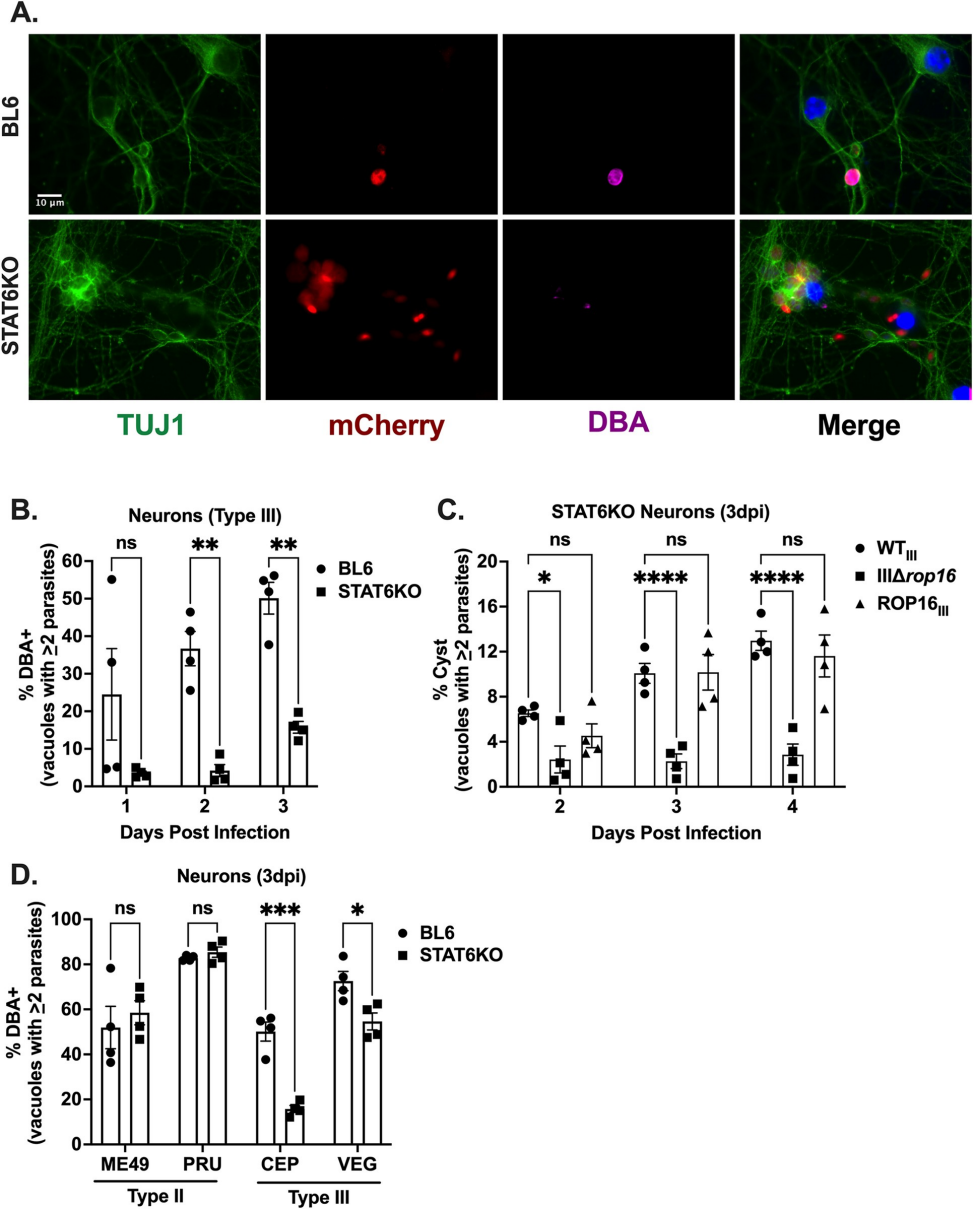
STAT6 facilitates encystment of type III, but not type II parasites, *in vitro*. (A) IFA of cyst assay. BL6 (control) or STAT6KO PNCs were infected with WT_III_ (MOI 0.1) for 2 dpi. Images depict anti-TUJ1 (green, neurons), mCherry (red, parasites), DBA (magenta), and DAPI (blue). Scale bar = 10μm. (B) Quantification of encystment of WT_III_ parasites overtime in identified PNCs. (C) Quantification of encystment of WT_III_, IIIΔ*rop16*, and ROP16_III_ parasites overtime in STAT6KO PNCs. (D) Quantification of encystment in identified PNCs at 3 dpi for listed type II and type III strains. (B-D) Bars, mean ± SEM. N = 5 wells/experiment, 4 experiments total. (B, D) *p≤0.05, **p≤0.005, ***p≤0.0005, ns = not significant, two-way ANOVA, Dunnett’s multiple comparisons test compared to BL6. (C) *p ≤ 0.05, ****p≤0.0001, ns = not significant, two-way ANOVA, Dunnett’s multiple comparisons test compared to WT_III_.

As the STAT6 knock-down showed the strongest effect on cyst formation and STAT6KO mice are commercially available, we decided to test the role of STAT6 in cyst development using PNCs from STAT6KO mice [39]. We observed a significant decrease in encystment of WT_III_ parasites in STAT6KO PNCs at all time points compared to parasites in PNCs from BL6 control mice (**Fig 4A and 4B**). To determine if the type III ROP16 encystment defect was solely mediated by STAT6, we infected STAT6KO PNCs with WT_III_, IIIΔ*rop16*, or the ROP16_III_ complement and tracked cyst formation over time. Compared to WT_III_ or ROP16 complemented parasites, the IIIΔ*rop16* mutant continued to show a significant decrease in cyst formation (**Fig 4C**). Finally, to determine if host cell STAT6 was required for efficient encystment in other *T*. *gondii* strains, we infected STAT6KO PNCs with two type II strains (ME49 and PRU/WT_II_) and two type III strains (CEP/WT_III_ and VEG). At 3 dpi neither of the type II strains showed a defect in encystment, while both type III strains displayed a statistically significant decrease in encystment in STAT6KO PNCs (**Fig 4D**). Taken together these results indicate that STAT6 enhances cyst development in HFFs and PNCs for type III strains, but not type II strains. In addition, the data suggest that other targets of ROP16 (e.g., STAT5a) also play a role in cyst formation.

### STAT6 is required for maximal encystment of type III parasites *in vivo*

**Fig 5:**
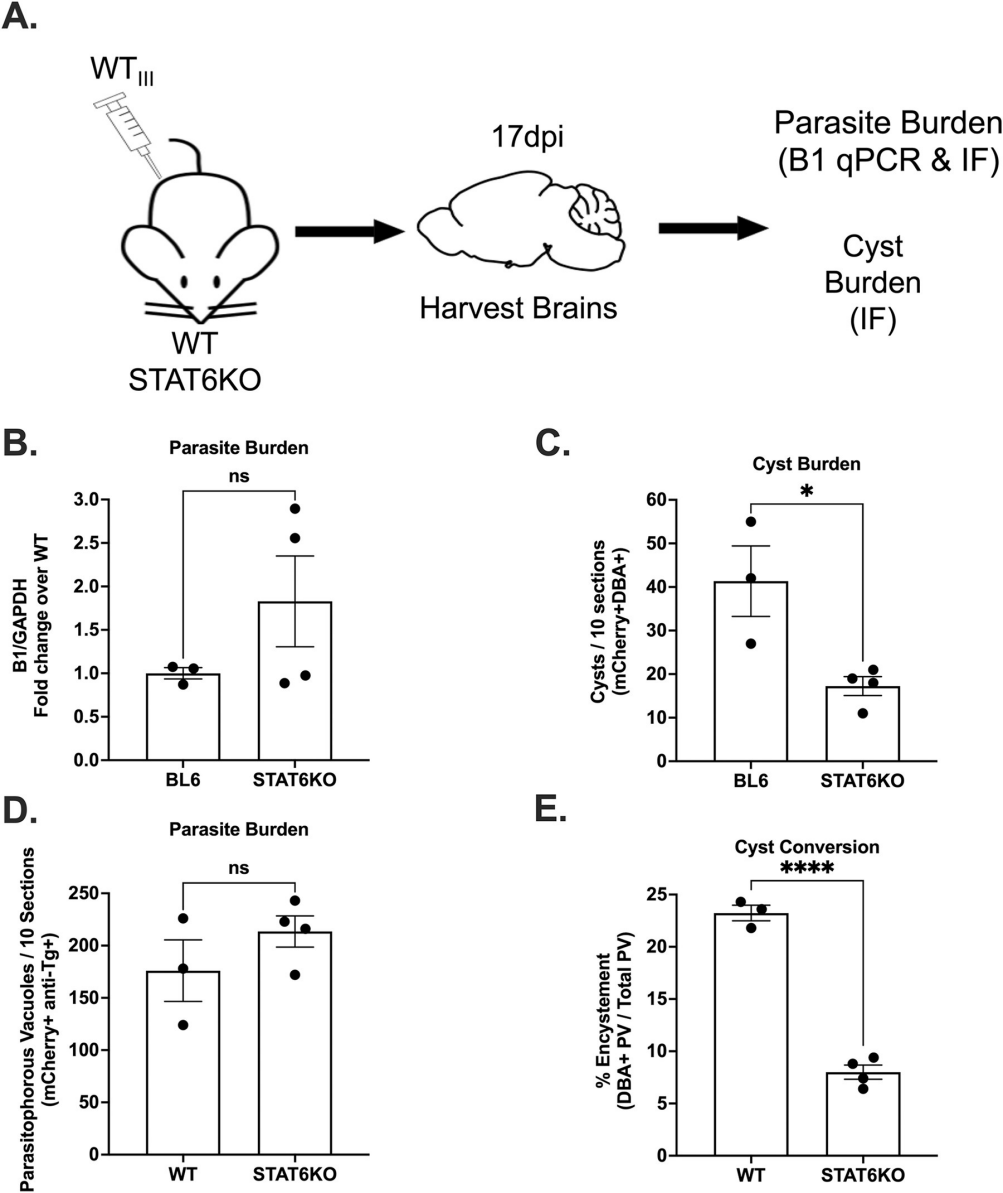
STAT6KO mice have lower CNS cyst burdens, but not overall CNS parasite burden, at 17 dpi. (A) Schematic of mouse infection for STAT6KO mice. (B) Quantification of *T*. *gondii* gene B1 was done using qPCR. Bars, mean ± SEM. Black dots = 1 mouse. N = 3–4 mice per condition. (C) Quantification of cyst burden (mCherry^+^DBA^+^). Total number of cysts per 10 sections per mouse. (D) Quantification of the parasitophorous vacuole (PVs) burden. Total number of PVs per 10 sections per mouse. (E) Quantification of the percentage of total PVs that were also DBA^+^. (B-E) Bars, mean ± SEM. Black dots = 1 mouse. N = 3–4 mice per condition. *p≤0.05., p****≤0.0001, ns = not significant, one-way ANOVA, Dunnett’s multiple comparisons test compared to BL6.

As previously noted, deletion of ROP16 from type III strains causes the parasite to be cleared during acute infection which results in a significant decrease in parasite dissemination to the CNS and thus cyst burden [36,37], making it difficult to assess the role of ROP16 during latent infection *in vivo*. However, the identification of STAT6 as a downstream mediator of the ROP16 encystment phenotype *in vitro* opened an avenue for assessing the role of STAT6 during latent infection *in vivo*. To test the role of STAT6 in latent infection, we infected STAT6KO mice and BL6 control mice with the WT_III_ parasites and harvested brains at 17 dpi (**Fig 5A**). When we quantified parasite burden by quantitative PCR (qPCR) for the *T*. *gondii*-specific gene B1, we observed no significant difference between STAT6KO and BL6 control mice (**Fig 5B**). In contrast, when we quantified cyst burden via immunofluorescent staining of infected CNS tissue (mCherry^+^DBA^+^), we observed a significant decrease in CNS cyst burden in STAT6KO mice compared to BL6 mice (**Fig 5C**). To confirm that this discrepancy was secondary to differences in encystment, we stained tissue sections with the polyclonal anti-*T*. *gondii* antibody that stains all PVs as well as DBA. We then quantified the number of parasite PVs (anti-*T*. *gondii* antibody^+^mCherry^+^) and the percentage of PVs that were also DBA^+^ (antibody^+^mCherry^+^DBA^+^). Consistent with the B1/DBA discrepancy, we found similar numbers of total PVs in both BL6 and STAT6KO mice (**Fig 5D**) but STAT6KO mice had significantly fewer DBA^+^ PVs (**Fig 5E**).

**Fig 6:**
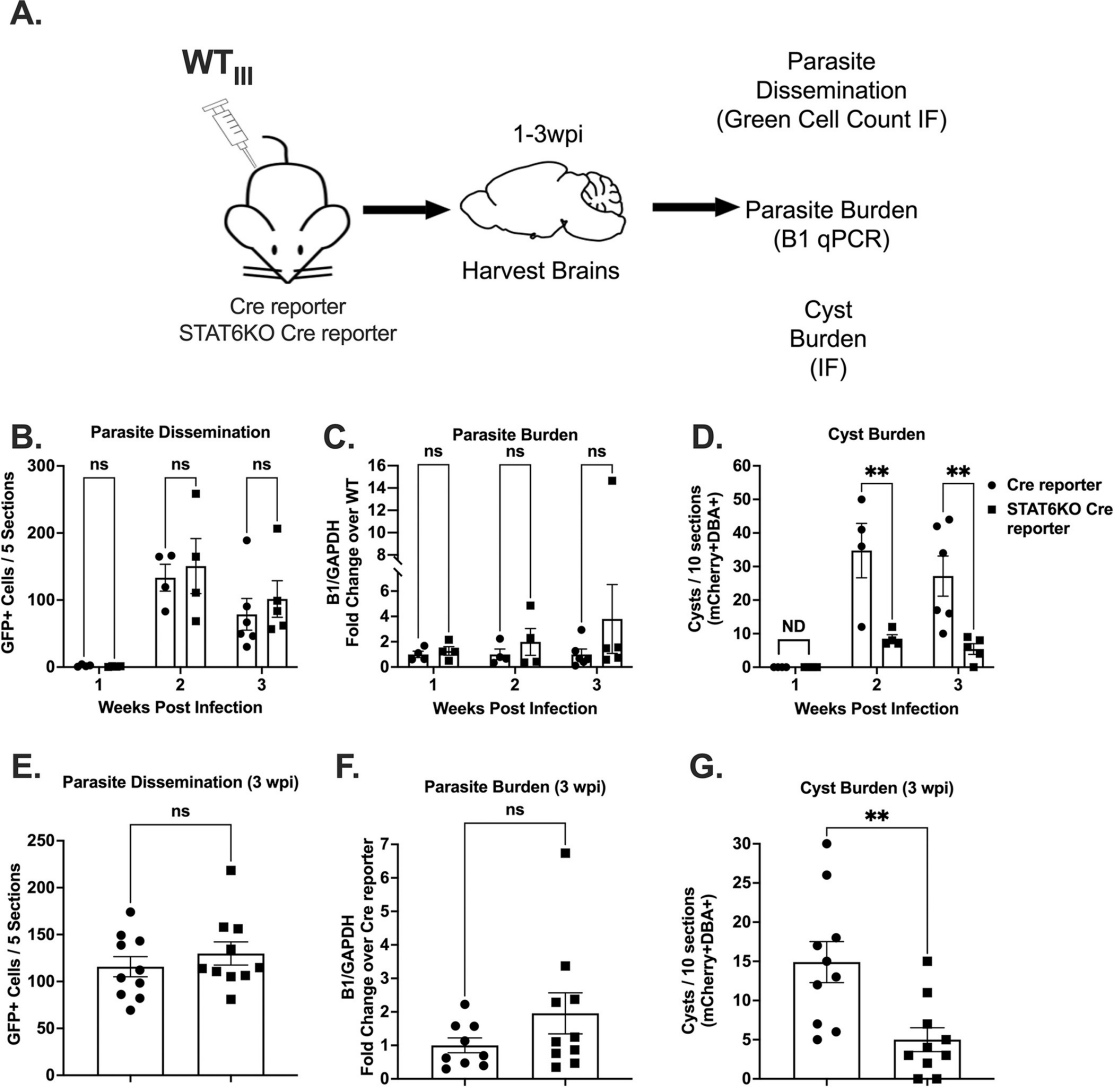
STAT6KO mice consistently have lower cyst burdens despite similar CNS parasite dissemination and overall parasite burden. (A) Schematic of mouse infection for Cre reporter (control) and STAT6KO Cre reporter mice. (B) Quantification of the total number of GFP^+^ cells per 5 sections per mouse. (C) Quantification of *T*. *gondii* gene B1 as in (**Fig 5B**). (D) Quantification of cyst burden as in (**Fig 5C**). (E) Quantification of the total number of GFP^+^ cells per 5 sections per mouse. (F) Quantification of *T*. *gondii* gene B1 as in (**Fig 5B**). (G) Quantification of cyst burden as in (**Fig 5C**). (B-G) Bars, mean ± SEM. Black dots = 1 mouse. **p≤0.005, ns = not significant, two-way ANOVA, Dunnett’s multiple comparisons test compared to Cre-reporter. (B-D) N = 4–6 mice per condition per timepoint. (E-G) N = 9–10 mice per condition.

We next sought to determine if our cyst defect was secondary to differences in parasite dissemination to the CNS. To address this possibility, we generated STAT6KO Cre reporter mice by crossing STAT6KO mice to Cre reporter mice [52] that express a green fluorescent protein (GFP) only after the cells have undergone Cre-mediated recombination (**S6 Fig**). We infected control Cre reporter mice and STAT6KO Cre reporter mice with the WT_III_ parasites as they also express Cre recombinase fused to a parasite protein [40,53] that is injected into the host cell before invasion. As this system allows us to mark and track cells injected with *T*. *gondii* protein, we use the quantification of GFP^+^ CNS cells as a proxy for parasite dissemination to the CNS. At 1–3 weeks post infection (wpi), we harvested brains to assess parasite dissemination to the CNS and CNS parasite and cyst burden (**Fig 6A**). There was no significant difference in the CNS GFP^+^ cell count at any timepoint between STAT6KO Cre reporter and control Cre Reporter mice (**Fig 6B**). Consistent with the prior cohort (**Fig 5B**), we observed no significant difference in CNS parasite burden between STAT6KO Cre reporter and control Cre reporter mice at any timepoint (**Fig 6C**). In contrast to the GFP^+^ cell count and qPCR data, we observed a significant decrease in the CNS cyst burden at 2 and 3 wpi in STAT6KO Cre reporter mice (**Fig 6D**). To confirm the reproducibility of the STAT6 encystment defect, we repeated the infection of STAT6KO Cre reporter and control Cre reporter mice for the 3 wpi time point. Consistent with the prior experiment, compared to control Cre reporter mice, STAT6KO Cre reporter mice had similar CNS GFP^+^ cell numbers and parasite burdens but lower cyst burdens (**Fig 6E–6G**). To determine whether STAT6 influenced the infectivity of type III cysts, we intraperitoneally infected STAT6KO Cre reporter mice and control Cre reporter mice with WT_III_ parasites. At 3 wpi, brains were harvested from the appropriate mice and fed to CBA/j mice. At 3 wpi, all CBA/j mice had similar CNS parasite burdens (**S7 Fig**).

Finally, to determine how a loss of STAT6 affected type II parasites *in vivo*, we infected STAT6KO Cre reporter and control Cre reporter mice with WT_II_ parasites which also express the Cre recombinase fusion protein[51]. At 3 wpi, we harvested brains to assess for parasite dissemination, parasite burden, and cyst burden. We saw no difference in WT_II_ dissemination to the CNS, over all CNS parasite burden, or cyst burden, indicating that a lack of STAT6 does not affect encystment of type II parasites *in vivo* (**S8 Fig**), at least at this time point. Together these results highly suggest that, for type III parasites but not type II parasites, STAT6 influences encystment *in vivo*, but does not affect parasite dissemination or overall CNS parasite burden up to 3 wpi.

### ROP16 also enhances encystment in human neurons

**Fig 7:**
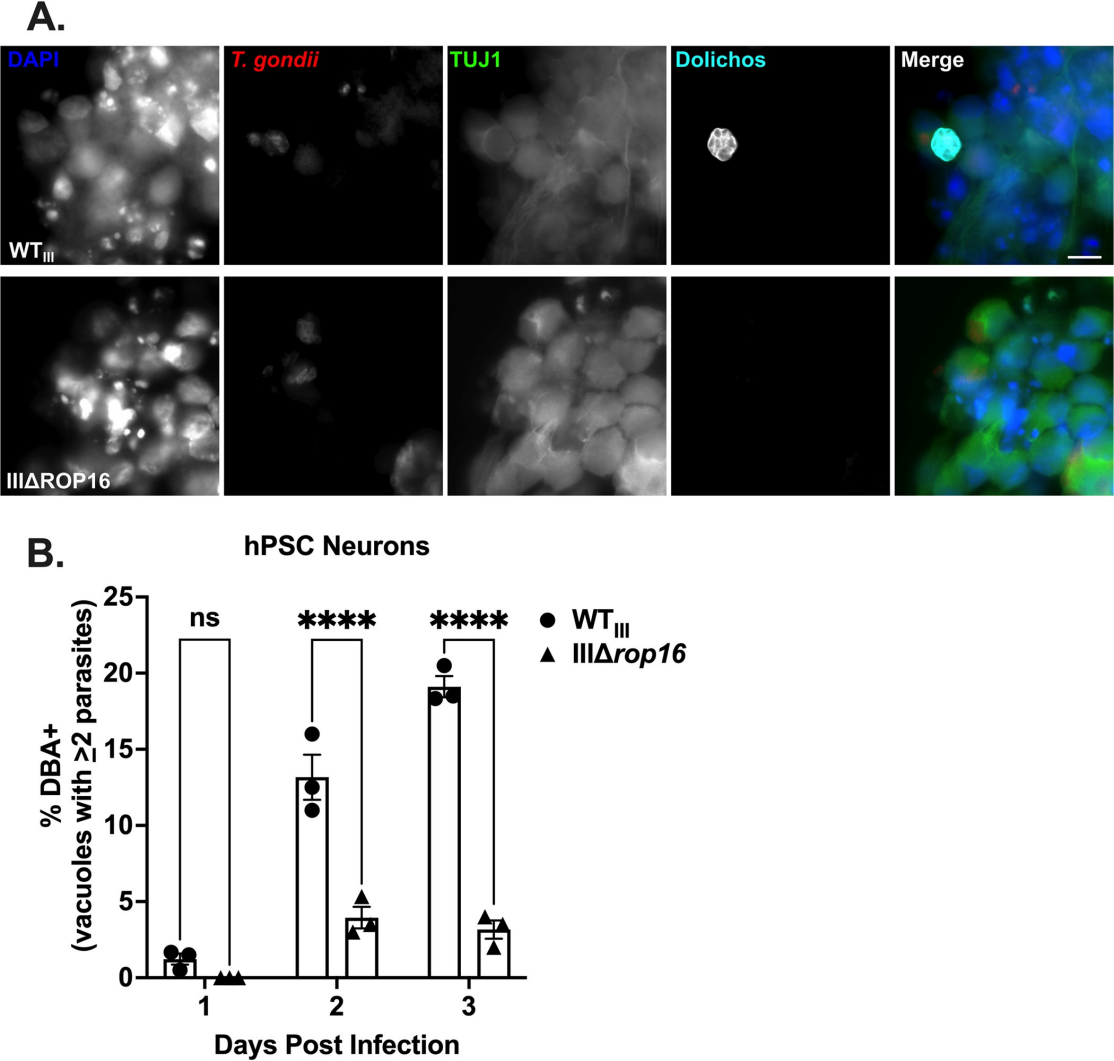
Deletion of ROP16 impairs cyst development in human neurons. (A) IFA of infected (3 dpi) human stem cell derived neurons (hPSC neurons). Images depict anti-TUJ1 (green, neurons), mCherry (red, parasites), DBA (cyan), and DAPI (blue). (B) Quantification of encystment at 1–3 dpi in hPSC neurons. Bars, mean ± SEM. N = 3 replicates/experiment, 3 independent experiments. ****p≤0.0001. ns = not significant, two-way ANOVA, Dunnett’s multiple comparisons test compared to WT_III_. Scale bar = 10μm.

As the prior work primarily defined the role of ROP16 in murine neurons and the mouse CNS but human toxoplasmosis is the disease we ultimately seek to understand, we sought to determine how ROP16 affected cyst development in human neurons. To address this possibility, we differentiated human pluripotent stem cells into neurons (hPSC neurons) and then infected them with WT_III_ or IIIΔ*rop16* parasites for 1–3 days. We then quantified the percentage of encysted vacuoles over time using immunofluorescent staining with DBA (**Fig 7A**). In addition to an overall reduction in encystment in hPSC neurons compared to murine PNCs, we saw a significant defect in encystment (~66% reduction) at 2 and 3 dpi for IIIΔ*rop16* parasites compared to WT_III_ parasites (**Fig 7B**). These results suggest that, like murine neurons, ROP16 facilitates encystment of type III parasites in human neurons. These results also suggest that *T*. *gondii* strains may show different propensities for encystment between human and murine neurons.

## Discussion

The data presented here show that, in human and murine *in vitro* models of encystment and in mice *in vivo*, ROP16 mediates efficient encystment of type III parasites, in part, via activation of STAT6. To the best of our knowledge, these data are the first to identify a host gene that shows a strain-specific effect on encystment. The importance of this latter point is that it questions the assumption that encystment is a process largely conserved between *T*. *gondii* strains.

The *in vitro* work emphasizes that parasite-host cell outcomes often vary by cell type and context. In non-neuronal cells, ROP16-mediated activation of STAT6 has been shown to influence tachyzoite survival by suppressing IFN-γ-induced nitric oxide production and IFN-γ-independent ROS production [31,38]. The data presented here show that in conditions that favor encystment (HFFs grown under alkaline stress and neurons), activation of STAT6 promotes parasite stage conversion. These ROP16-dependent, STAT6-mediated effects are presumptively driven by STAT6 altering gene expression in a cell-specific and context-dependent manner. Future studies will focus on defining the STAT6-dependent neuronal genes that promote encystment of type III strains.

While the *in vitro* data strongly suggest that the ROP16-dependent encystment phenotype is driven by STAT6 in the host cell, the *in vivo* data cannot distinguish between a direct or indirect effect of STAT6 signaling because we used a global STAT6 knockout [39]. For example, the lack of STAT6 signaling in immune cells may lead to changes in levels of specific cytokines or chemokines in the CNS, which may indirectly influence the rate or timing of type III encystment in neurons. Such possibilities are relevant in this context because *T*. *gondii* activation of STAT6 signaling has been shown to influence macrophage polarization [32,37], T cell activation [37,54], and arginase 1-dependent limitations on parasite growth rate in peritoneal macrophages [31]. Distinguishing between direct and indirect effects of STAT6 signaling on type III encystment *in vivo* will require future studies that use cell-specific STAT6 knockouts and/or bone marrow chimeras in combination with examination of encystment dynamics over a longer time.

While STAT6 plays an important role in ROP16-dependent type III encystment, the data showing that III*Δrop16* parasites have decreased encystment in STAT6KO neurons compared to WT_III_ parasites (**Fig 4C**) suggests that another host factor is involved in this phenotype. As HFFs with decreased levels of STAT5a also show an encystment defect (**S5 Fig**), STAT5a maybe the other host factor. Of course, this host factor might also be a completely novel ROP16 substrate. Fully defining the host factors/pathways involved in the ROP16-dependent type III encystment will be the focus of future studies.

The identification of a role for ROP16 in cyst development also raises the question of what other *T*. *gondii* secreted effector proteins prime cells to facilitate encystment. Until now, *T*. *gondii’s* repertoire of effector proteins have largely been studied in tachyzoite conditions and during acute infection. Much less is known about how *T*. *gondii*’s manipulation of host cells influences other stages of the parasite’s lifecycle. As ROPs are largely thought to be secreted only during the initial invasion process [55], any effects on cyst development are likely to be involved in priming the host cell to influence the early events of stage conversion. The dense granule proteins (GRAs) are known to be secreted post invasion [56], with evidence suggesting that at least one of these GRAs (TgIST) is secreted post cyst formation [57]. Thus, these effectors may play a larger role in cyst development and maintenance. As seen in this work, understanding how *T*. *gondii* effectors influence cyst development may provide a mechanism for identifying more host cell pathways and proteins that enable or trigger encystment.

Finally, ROP16 appears to be dispensable for encystment of type II parasites, indicating that there are strain-specific differences in stage conversion and cyst development. Prior strain-specific differences in stage conversion have been observed and were linked to parasite replication rates [58,59]. The data presented here suggest an additional level of complexity—different *T*. *gondii* strain types may require strain-specific host cell manipulations to produce optimal bradyzoite-inducing conditions. That type II strains lack a ROP16 that activates STATs and thus do not cause prolonged STAT6 activation [30,34] raises multiple questions such as: do type II strains have unique host cell requirements for optimal encystment? Or have they developed alternative mechanisms to activate the same downstream, encystment-promoting host genes? The availability F1 progeny from the type II x type III cross [25,30] in combination with our newly developed automated cyst imaging protocol opens a way to use quantitative trait loci mapping as a powerful tool to identify more parasite genes and associated host pathways that influence strain-specific differences in encystment.

In summary, the data presented here show that ROP16-dependent activation of STAT6 mediates efficient encystment of type III, but not type II, *T*. *gondii*. These findings highlight that the outcome of host cell manipulation varies by host cell type and context. They also suggest that there are multiple layers of control for achieving maximal encystment and that these layers may vary between *T*. *gondii* strains.

## Methods

### Ethics statement

All procedures and experiments were carried out in accordance with the Public Health Service Policy on Human Care and Use of Laboratory Animals and approved by the University of Arizona’s Institutional Animal Care and Use Committee (#12–391). All mice were bred and housed in specific-pathogen-free University of Arizona Animal Care facilities.

#### Parasite maintenance

As previously described, all parasite strains used in this study were maintained through serial passage in human foreskin fibroblasts (gift of John Boothroyd, Stanford University, Stanford, CA) using DMEM, supplemented with 10% fetal bovine serum, 2 mM glutagro, and 100 IU/ml penicillin and 100 μg/ml streptomycin.

#### Parasites strains

Except for the strains identified in Fig 4D, all type III strains are derived from CEP and all type II strains are derived from Prugniaud/PRU. Unless otherwise specifically noted, WT_III_ (CEP) and WT_II_ (PRU) express mCherry and a rhoptry::Cre recombinase fusion protein and have been previously described [40,51]. The generation of IIIΔ*rop16* and ROP16 mutant parasites, all of which express tdTomato, were previously described [38]. The WT_III_::GFP strain expresses GFP and firefly luciferase and was made in a CEP strain that is resistant to Ara-C and sinefungin [60] (gift of Jon Boyle, University of Pittsburgh, Pittsburgh, Pennsylvania). VEG and ME49 strains have been previously described [44,61] (gift of Jon Boyle, University of Pittsburgh, Pittsburgh, Pennsylvania). For the IIΔ*rop16* strain, type II parasites (PRUΔ*hxgprt*) (gift of Dominique Soldati-Favre, University of Geneva, Geneva, Switzerland) were co-transfected with the sgROP16Up CRISPR and sgROP16Down CRISPR plasmids along with the pTKO plasmid containing ROP16 homology regions surrounding a hypoxanthine phosphoribosyltransferase (*hxgprt)* cassette [36]. Parasites were then screened for expression of *hxgprt* using media containing 25 mg/ml mycophenolic acid and 50 mg/ml xanthine, prior to cloning by limiting dilution [62].

#### Mice

Cre reporter mice (strain no. 007906) and STAT6KO mice (strain no. 005977) were originally purchased from Jackson Laboratories. STAT6KO Cre reporter homozygotes were generated by breeding STAT6KO and Cre reporter mice and resulting heterozygotes progeny. The homozygosity at both loci were confirmed by PCR (**S6 Fig**). Mice were inoculated with 10,000 freshly syringe-lysed parasites diluted in 200 μl of USP grade phosphate buffered saline (PBS). Age- and sex-matched mice were used. For brain feeding, CBA/j mice were purchased from Jackson Laboratories (strain no. 000654), fasted overnight, and then placed in individual cages and offered whole brain. The mice remained in individual cages until each brain was eaten (<3 hours).

#### Primary murine neuron culturing

Mouse primary cortical neurons were harvested from E17 mouse embryos obtained from pregnant Cre reporter or STAT6KO mice. Dissections of E17 cortical neurons were performed as described previously. Primary neuronal cell cultures were generated by methods described previously with minor modifications [63]. Culture plates were prepared by coating with 0.001% poly-L-lysine solution (Millipore Sigma, P4707, diluted in water 1:10) for plastic surfaces and 100 μg/ml poly-L-lysine hydrobromide (Sigma, P6282, dissolved in borate buffer, pH 8.4) for glass surfaces overnight. They were washed three times for 10 minutes each with water and transferred to plating media (MEM, 0.6% D-glucose, 10% FBS). Neurons were seeded at appropriate densities: 100,000 in 24 well plates with coverslips for confocal imaging and 20,000 in 96 well plates for use on the Operetta platform. Four hours after plating, full media exchange to neurobasal media (Neurobasal base media, 2% B27 supplement, 1% L-glutamine and 1% penicillin-streptomycin) was performed. On day in vitro (DIV) 4, neurons received a half volume media change of neurobasal media with 5 μM cytosine arabinoside (AraC, Millipore Sigma, C6645) to stop glial proliferation. One third media exchanges with neurobasal media occurred every 3–4 days thereafter. All experiments were performed on 10 DIV neurons.

#### Cyst assays

Confluent HFF monolayers and primary neurons were cultured on glass coverslips, 96 well plates (Perkin Elmer; 6055302), or 6 well plates as indicated and infected with parasites at the MOIs indicated in the figure legends. For the alkaline stress model [10], HFFs were infected with parasites for 4 hours under non-cyst inducing conditions (pH 7.1 and CO_2_ = 5%) to allow the parasites to invade. After 4 hours, the media was changed to cyst inducing conditions (50 mM HEPES [pH 8.1] in RPMI supplemented with 1% fetal bovine serum, penicillin, and streptomycin). Media changes occurred every 2 days to maintain pH levels. For primary neuronal cell cultures, parasites were simply added to culture at the indicated MOIs. For Operetta analyses, entire wells were imaged at 20x (69 total fields of view). 5–10 wells were imaged per experiment depending on the assay. Cells were fixed at the indicated timepoints and stained as described below. Images were then analyzed with Harmony software. Briefly, host cell nuclei were identified and enumerated via DAPI staining and the standard nuclei identification module. PVs were stained using an antibody that stains the PVM (Invitrogen PA17252). Stained PVs were identified using the Harmony find spots tool. Fluorescent intensity cutoffs were set at 10,000 and length and width cutoffs were set at 5 μm and 2 μm respectively. For purposes of cyst quantification, we wanted to exclude vacuoles with fewer than 2 parasites as these could be recent infections that have not yet had time to form cysts. To identify vacuoles with ≥2 parasites length and width cutoffs were set at 8 μm and 4 μm respectively. To identify cysts, we used the identify image area tool on DBA^+^ PVs identified as having ≥2 parasites. The intensity cutoff for positive cyst staining was set at 7,500 as determined by taking the average DBA background signal for parasites stained under non-cyst inducing conditions. The percent encystment in each well was then calculated by taking the number of DBA^+^ PVs (≥2 parasites) divided by the total number of PVs (≥2 parasites) followed by multiplication by 100. All Harmony Analysis programs are available upon request.

#### Co-infection cyst assay analysis

Confluent HFF monolayers were cultured on glass coverslips and infected with parasites at an MOI of 1 with either WT_III_::GFP parasites alone, IIIΔ*rop16* alone or WT_III_::GFP and IIIΔ*rop16* at a 1:1 ratio. HFFs were infected with parasites for 4 hours under non-cyst inducing conditions (pH 7.1 and CO_2_ = 5%) to allow the parasites to invade. After 4 hours, the media was changed to cyst inducing conditions (50 mM HEPES [pH 8.1] in RPMI supplemented with 1% fetal bovine serum, penicillin, and streptomycin). Media changes occurred every 2 days to maintain pH levels. To identify cysts, we stained parasites with Biotinylated-DBA at 1:500, followed by incubation with Strepavadin-647 (1:500). For single infections percent encystment was calculated by counting GFP+DBA+ for WT_III_::GFP or tdTomato+DBA+ for IIIΔ*rop16* divided by the total number of GFP+ or tdTomato+ parasites multiplied by 100. For co-infections we scored encystment for only the IIIΔ*rop16* parasite which were in a host cell with WT_III_::GFP. Using a standard epifluorescent microscope (EVOS), we counted 100 vacuoles per coverslip, 3 coverslips/experiment.

#### Immunofluorescence microscopy

For immunofluorescence assays, cells were fixed with 4% paraformaldehyde for 15 min. The cells were then permeabilized and blocked for 60 min with 0.1–0.2% (vol/vol) Triton-X 100 in PBS (pH 7.4), typically with 3% (wt/vol) goat serum. For Operetta analyses and confocal microscopy of type II parasites (**S4 Fig** top) in the alkaline-stress induced model of encystment, cells were incubated overnight with Biotinylated-DBA at 1:500 and rabbit anti-*T*. *gondii* polyclonal antibody (Invitrogen PA17252) at 1:10,000, followed by incubation with Strepavadin-647and anti-rabbit Alexa Fluor-488 for 1 hour. For Operetta analyses and confocal microscopy of type II parasites (**S4 Fig** bottom) in PNCs cells were stained with mouse anti-TUJ1 (Millipore Sigma, MAB1637) at 1:500, rabbit anti-*T*. *gondii* polyclonal antibody (Invitrogen PA17252) at 1:10,000 and Biotinylated-DBA at 1:500, followed by incubation with Strepavadin-647, anti-mouse Alexa Fluor-488, and anti-rabbit Alexa Fluor-568 secondary antibodies at 1:500 (Molecular Probes) for 1 hour. For IFA in **S1 Fig**, cells were incubated overnight with Biotinylated-DBA at 1:500; mouse anti-SAG1 (DG52) [41] at 1:10,000 (gift of John Boothroyd, Stanford University, Stanford, CA); and rabbit anti-SRS9 [64] at 1:10,000 (gift of John Boothroyd, Stanford University, Stanford, CA); followed by incubation with Strepavadin-647, anti-mouse Alexa Fluor 555, and anti-rabbit Alexa Fluor-488 at 1:500 (Molecular Probes) for 1 hour. For IFA in **S3 Fig**, cells were treated as above except only using anti-Tuj1 antibodies and biotinylated DBA. For 96 well plates (Operetta analyses), wells were covered with 100μLs of Fluoromount-G (SouthernBiotech 0100–01) diluted 1:50 in 1x PBS. Coverslips were mounted on slides using Fluoromount-G.

#### Tissue preparation for histology and DNA extraction

At appropriate times post infection, mice were anesthetized with ketamine (24 mg/ml) and xylazine (4.8 mg/ml) cocktail and transcardially perfused with ice cold PBS. After harvesting organs, the left half of the mouse brain was fixed in 4% paraformaldehyde in phosphate buffer and kept at 4°C overnight, rinsed in PBS, and then was embedded in 30% sucrose. Post fixation, brains were sectioned into 40 μm thick sagittal sections using a freezing sliding microtome (Microm HM 430). Sections were stored in the cryoprotective media (0.05 M sodium phosphate buffer containing 30% glycerol and 30% ethylene glycol) as free-floating sections until stained and mounted on to slides. The right half of the brain was sectioned coronally into 2 halves and stored in separate tubes. These tubes were flash frozen and stored at -80°C until used for DNA extraction.

#### Quantitative real time PCR

For quantification of parasite burden, genomic DNA from the rostral quarter of the frozen brain was isolated using DNeasy Blood and Tissue kit (69504, Qiagen) and following the manufacture’s protocol. The *T*.*gondii*-specific, highly conserved 35-fold repeat B1 gene was amplified using SYBR Green fluorescence detection with the Eppendorf Mastercycler ep realplex 2.2 system. GAPDH was used as control to normalize DNA levels. Results were calculated as previously described^36^.

#### Green cell, cyst counts, and encystment rates

For enumeration of CNS cells that had undergone Cre-mediated recombination, sagittal brain sections were washed and mounted as previously described [36]. The total number of GFP^+^ cells were enumerated by using a standard epifluorescent microscope (EVOS microscope). Analyses were performed on 5 brain sections per mouse, after which the resulting numbers were then averaged to obtain the mean number of GFP^+^ cells/section/mouse. For enumeration of cysts, sagittal brain sections were washed and blocked in 3% Goat Serum in 0.3% TritonX-100/TBS for 1 hour. Sections were then incubated with biotinylated DBA (Vector Laboratories 1031, 1:500) for 24 hours, followed by incubation with 647 Streptavidin (Invitrogen, 1:500) for 1 hour. Sections were mounted as described above. The number of cysts were enumerated using a standard epifluorescent microscope (EVOS microscope). Only objects that expressed mCherry and stained for DBA were quantified as cysts. For total parasitophorous vacuoles (PVs) and encystment rates, sections were incubated with biotinylated DBA and rabbit anti-*T*. *gondii* polyclonal antibody (Invitrogen PA17252) at 1:1000 for 24hrs, followed by incubation with Strepavadin-647, and anti-rabbit Alexa Fluor 488 1:500 for 1 hour. Mounted sections were then analyzed using a standard epifluorescent microscope (EVOS microscope). PVs were identified by positivity for anti-*T*. *gondii* antibodies and mCherry expression (antibody^+^mCherry^+^). Only PVs that were also positive for DBA (antibody^+^mCherry^+^DBA^+^) were quantified as cysts. For GFP^+^ cell counts in WT_II_ infection, the same protocol outlined above was followed except only 3 sections per mouse were analyzed. For cyst counts in WT_II_ infection, the same procedure was followed as for encystment rates except that the rabbit anti-*T*. *gondii* polyclonal antibody was used with Alexa Fluor 568 as the secondary. Objects that were anti-*T*. *gondii* antibody^+^mCherry^+^DBA^+^ were quantified as cysts. For all analyses, investigators were blinded to the genotype of the mouse until after the data were collected.

#### Generation of hPSC neurons

H7 human embryonic neural stem cells (NSCs) derived from the NIH-approved H7 embyronic pluripotent stem cells (WiCell WA07) were purchased from the University of Arizona iPSC core (https://stemcells.arizona.edu/) and expanded on NSC expansion medium (NEM) (Thermofisher, Cat # A1647801) as previously described [65]. P2 passage NSCs were plated on poly-L-ornithine (20ug/ml) (Sigma, Cat # P4957) and laminin (5 ug/ml) (Thermo Fisher, Cat # 23017015) coated plates. For 14 days, the cells were differentiated into cortical neurons using neural differentiation medium (NDM) consisting of Neurobasal medium, 2 mM L-Glutamine, 1% B-27, 200 μM L- Ascorbic acid (Sigma, Cat # A92902), 0.5 mM c-AMP (Stem Cell Technologies Cat # 73886), 20 ng/ml BDNF (Stem Cell Technologies Cat # 78005), 20 ng/ml GDNF (Stem Cell Technologies Cat # 78058), 20 ng/ml NT-3 (Stem Cell Technologies Cat # 78074), and Penicillin/Streptomycin cocktail. The culture medium was exchanged with fresh NDM every 2–3 days.

#### Statistical analysis

Graphs were generated and statistical analyses performed using Prism 8.4.2 software. All experiments were performed at least three independent times, and statistical analyses were conducted on the composite data unless reported otherwise. Unless otherwise specified, the data were analyzed using a two-way analysis of variance (ANOVA) with Dunnett’s multiple comparisons test to the control (WT_III_ or WT_II_). For *in vivo* infections at a single timepoint a one-way ANOVA with Dunnett’s multiple comparisons test to the control (BL6) was used.

## Supporting information

S1 Fig ROP16_III_-deficient parasites are impaired in cyst development in alkaline stressed HFFs. IFA of cyst assay in HFFs. HFFs were infected with the indicated strains and subjected to alkaline stress and CO_2_ depletion for 6 days followed by fixation and staining as indicated. Images depict DBA (green), anti-SAG1 (red, tachyzoites), anti-SRS9 (magenta, bradyzoites), and DAPI (blue). Scale bar = 20μm.
(TIFF)

S2 Fig Parasite survival or number per PV do not appear to alter encystment rates in ROP16_III_ deficient parasites. (A) Quantification of accumulation of PVs over time in a stress model of encystment relative to 2 dpi. (B) Quantification of % encystment as a factor of number of parasites/vacuole at 2 dpi in a stress model of encystment. (C) Quantification of accumulation of PVs over time in a PNCs relative to 1 dpi. (D) Quantification of % encystment as a factor of number of parasites/vacuole at 2 dpi in PNCs. (A,C) Bars, mean ± SEM. Black dots = 1 experiment. N = 10 wells/ experiment, 3–4 experiments total. (B,D) Bars, mean ± SD. Black dots = 1 replicate. N = 3 replicates/experiment, 1 experiment.
(TIF)

S3 Fig ROP16_III_-deficient parasites are impaired in cyst development in PNCs. IFA of cyst assay in PNCs. PNCs were infected with the indicated strains for 2 days followd by fixation and staining as indicated. Images depict anti-TUJ1 (green, neurons), RFP (red, mCherry (WT_III_)/tdTomato (all others)), DBA (magenta), and DAPI (blue). Scale bar = 10μm.
(TIFF)

S4 Fig ROP16 is dispensable for cyst formation in type II parasites. Top, IFA of cyst assay in HFFs. HFFs were infected with the indicated strains for 2 days. Images depict anti-*T*. *gondii* staining (green), DBA (magenta), and DAPI (blue). Bottom, IFA of cyst assay in PNCs. PNCs were infected with the indicated strains for 2 days. Images depict anti-TUJ1 (green, neurons), anti-*T*. *gondii* (red), DBA (magenta), and DAPI (blue).
(TIFF)

S5 Fig Suppression of STAT6 consistently decreases WT_III_ encystment. Quantification of encystment at 2 and 4 dpi in alkaline stress model of encystment. Bars, mean ± SEM. N = 3 replicates/experiment, 3 experiments total. *p≤0.05 and **p≤0.005. ns = not significant, two-way ANOVA, Dunnett’s multiple comparisons test compared to shRNA non-targeting control (shNT).
(TIFF)

S6 Fig PCR confirmation of STAT6KO Cre reporter mice. Top, PCR for Cre reporter. Wild-type size = 297bp and mutant (Cre reporter) size = 199bp. Bottom, PCR for STAT6. Wild-type (STAT6) size = 275bp and mutant (STAT6KO) size = 380bp. + = positive control,— = negative control and nt = H_2_O control.
(TIFF)

S7 Fig WT_III_ cysts generated in STAT6KO background are still infectious. Cre reporter mice or STAT6KO mice were infected intraperitoneally with WT_III_ parasites. At 3 wpi, brains from these mice were fed to CBA/j mice. At 3 wpi, brains were harvested from CBA/js and CNS parasite burden quantified as in **Fig 5B**.
(TIFF)

S8 Fig STAT6KO does not alter parasite dissemination or cyst burden during type II infection. Cre reporter (control) and STAT6KO Cre reporter mice were infected and analyzed at 3 wpi as in **Fig 6** except using WT_II_ parasites and staining sections for cyst burden with anti-*T*. *gondii* antibody as well. *Left*, quantification of the total number of GFP^+^ cells per 3 sections per mouse as in **Fig 6A**. *Middle*, quantification of *T*. *gondii* gene B1 as in **Fig 5B.** *Right*, Quantification of cyst burden (anti-*T*. *gondii* antibody^+^mCherry^+^DBA^+^). Bars = mean. Black dots = 1 mouse. N = 4–5 mice per condition per cohort.
(TIFF)

S1 Data Excel spreadsheet with the raw numerical data for graphs in Figs 1–7, S2, S5, S7, and S8. Data for each graph is on individual sheets.
(XLSX)
