# Supplementary figures and images for: ROP16-mediated activation of STAT6 enhances cyst development of type III Toxoplasma gondii in neurons
Source: PLoS Pathog. 2023 Apr 17;19(4):e1011347. doi: 10.1371/journal.ppat.1011347 (PMC10138205; doi:10.1371/journal.ppat.1011347)

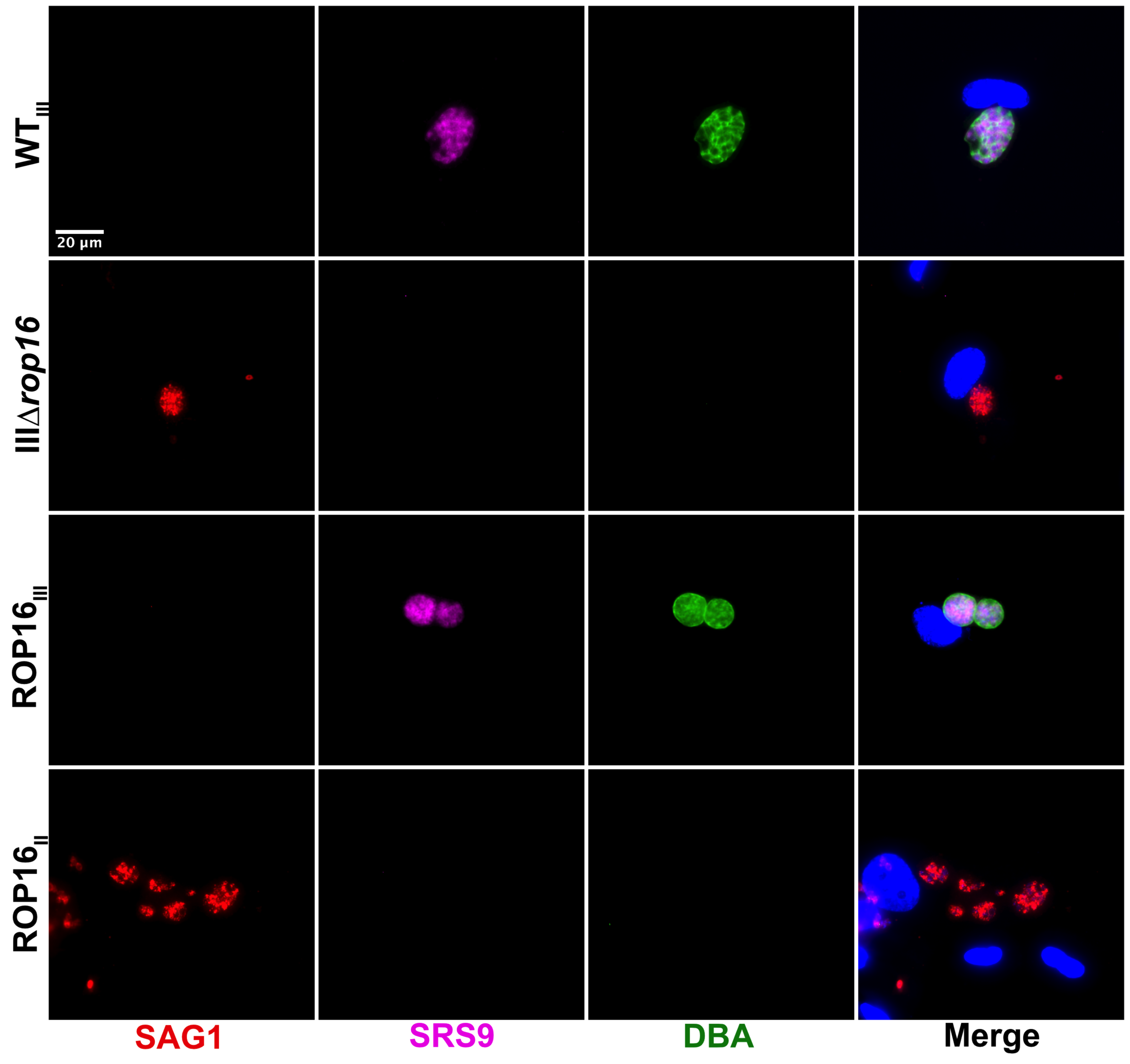

Supplement: S1 Fig — IFA of cyst assay in HFFs. HFFs were infected with the indicated strains and subjected to alkaline stress and CO2 depletion for 6 days followed by fixation and staining as indicated. Images depict DBA (green), anti-SAG1 (red, tachyzoites), anti-SRS9 (magenta, bradyzoites), and DAPI (blue). Scale bar = 20μm. (TIFF) [file ppat.1011347.s001.tiff]

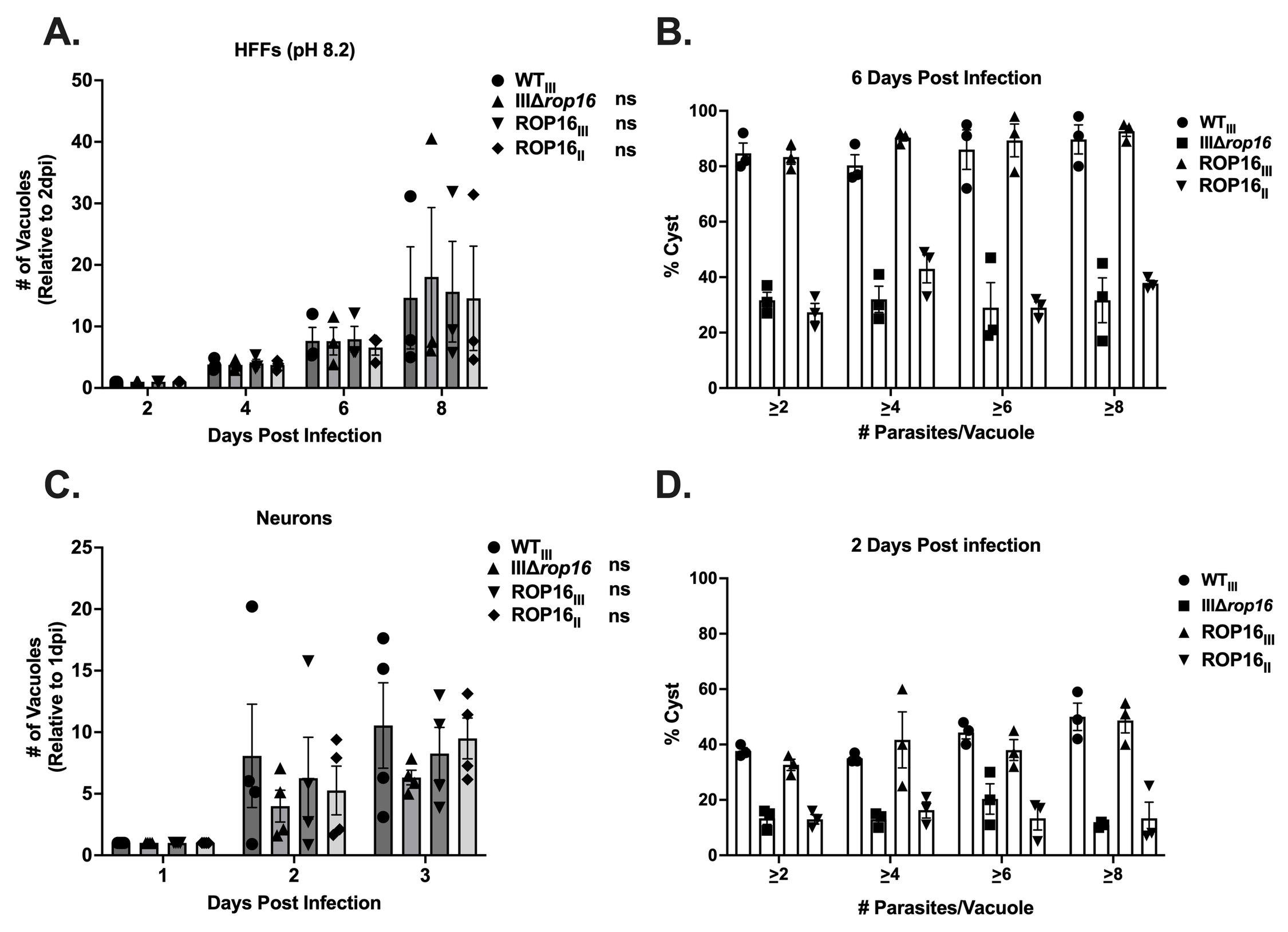

Supplement: S2 Fig — (A) Quantification of accumulation of PVs over time in a stress model of encystment relative to 2 dpi. (B) Quantification of % encystment as a factor of number of parasites/vacuole at 2 dpi in a stress model of encystment. (C) Quantification of accumulation of PVs over time in a PNCs relative to 1 dpi. (D) Quantification of % encystment as a factor of number of parasites/vacuole at 2 dpi in PNCs. (A,C) Bars, mean ± SEM. Black dots = 1 experiment. N = 10 wells/ experiment, 3–4 experiments total. (B,D) Bars, mean ± SD. Black dots = 1 replicate. N = 3 replicates/experiment, 1 experiment. (TIF) [file ppat.1011347.s002.tif]

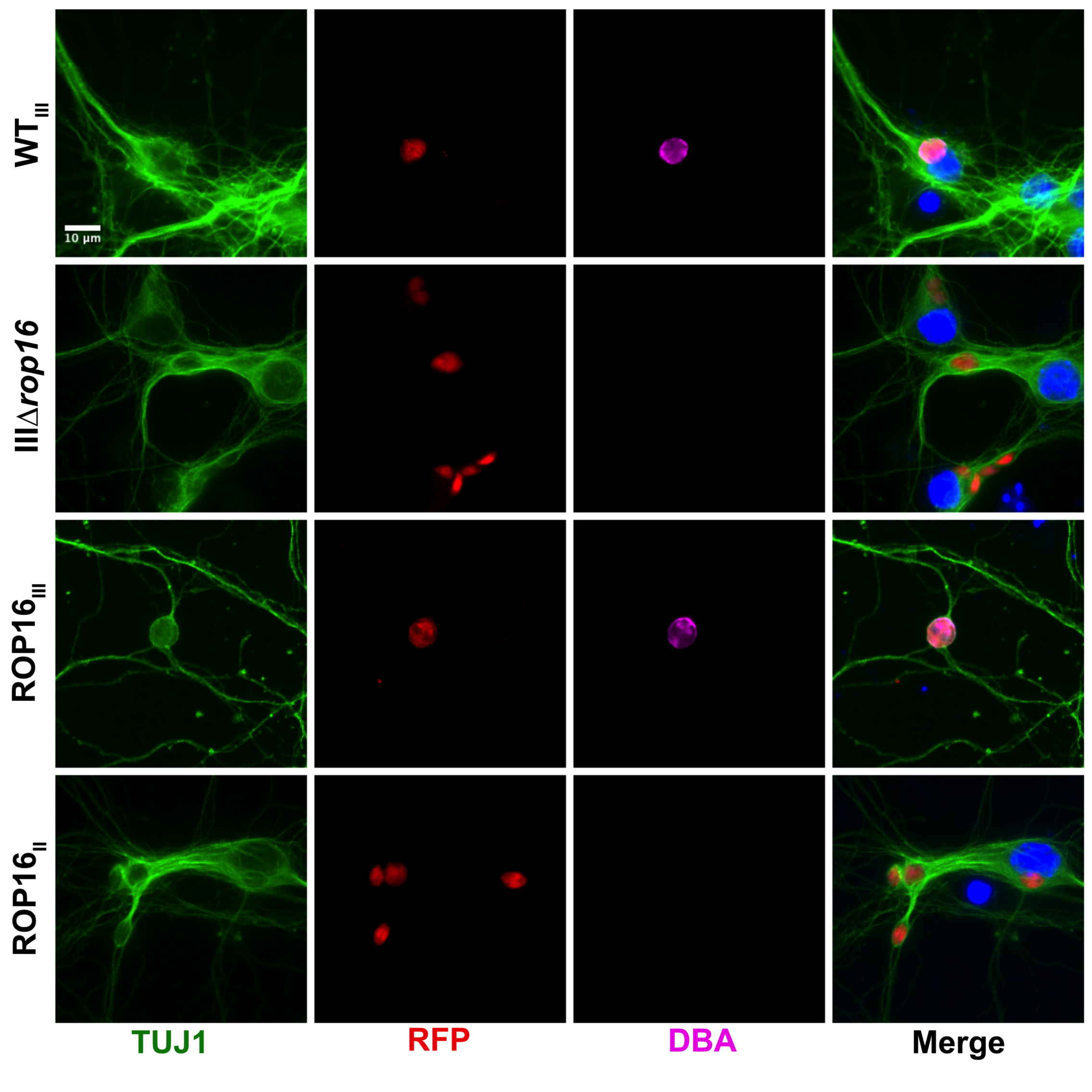

Supplement: S3 Fig — IFA of cyst assay in PNCs. PNCs were infected with the indicated strains for 2 days followd by fixation and staining as indicated. Images depict anti-TUJ1 (green, neurons), RFP (red, mCherry (WTIII)/tdTomato (all others)), DBA (magenta), and DAPI (blue). Scale bar = 10μm. (TIFF) [file ppat.1011347.s003.tiff]

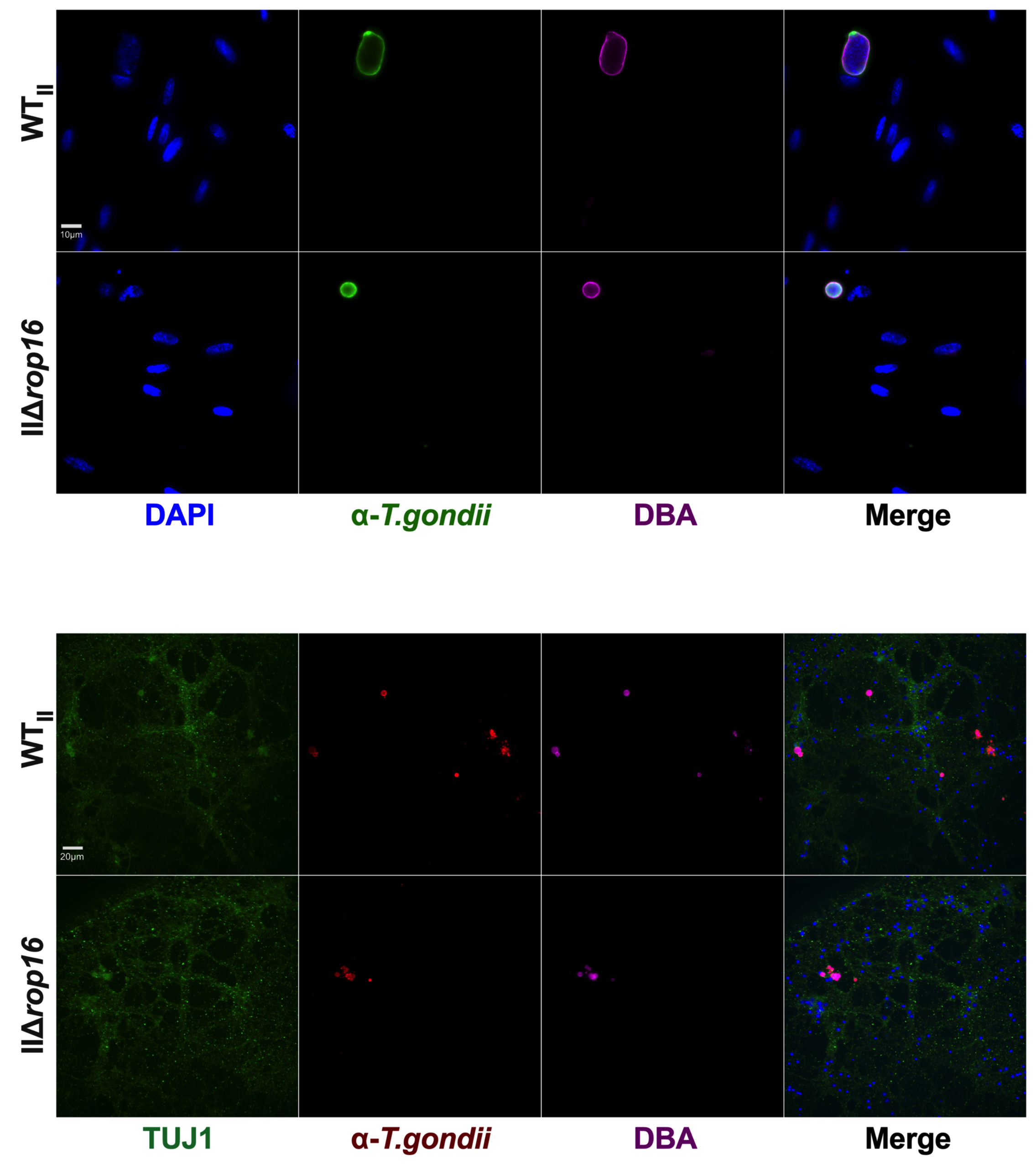

Supplement: S4 Fig — Top, IFA of cyst assay in HFFs. HFFs were infected with the indicated strains for 2 days. Images depict anti-T. gondii staining (green), DBA (magenta), and DAPI (blue). Bottom, IFA of cyst assay in PNCs. PNCs were infected with the indicated strains for 2 days. Images depict anti-TUJ1 (green, neurons), anti-T. gondii (red), DBA (magenta), and DAPI (blue). (TIFF) [file ppat.1011347.s004.tiff]

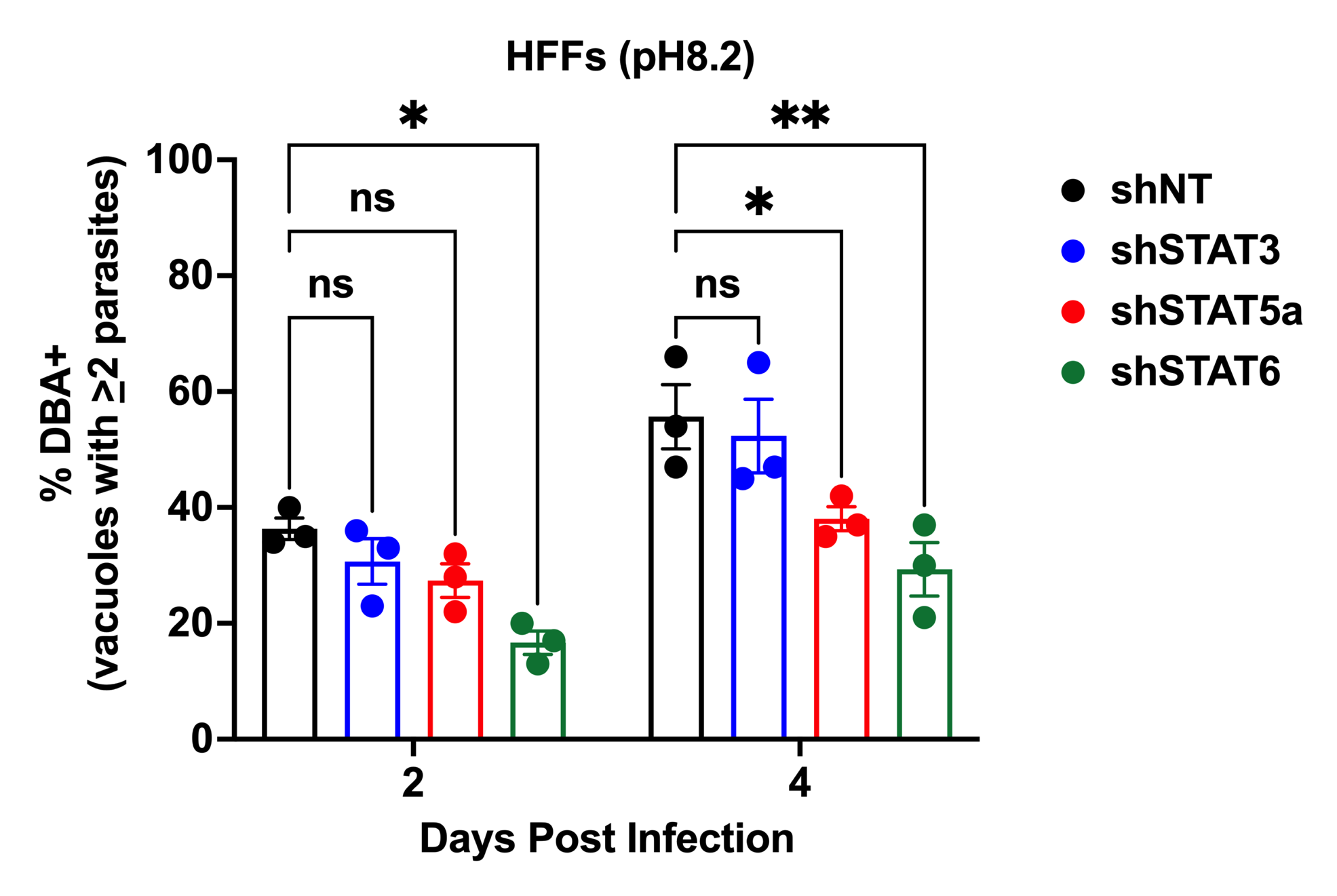

Supplement: S5 Fig — Quantification of encystment at 2 and 4 dpi in alkaline stress model of encystment. Bars, mean ± SEM. N = 3 replicates/experiment, 3 experiments total. *p≤0.05 and **p≤0.005. ns = not significant, two-way ANOVA, Dunnett’s multiple comparisons test compared to shRNA non-targeting control (shNT). (TIFF) [file ppat.1011347.s005.tiff]

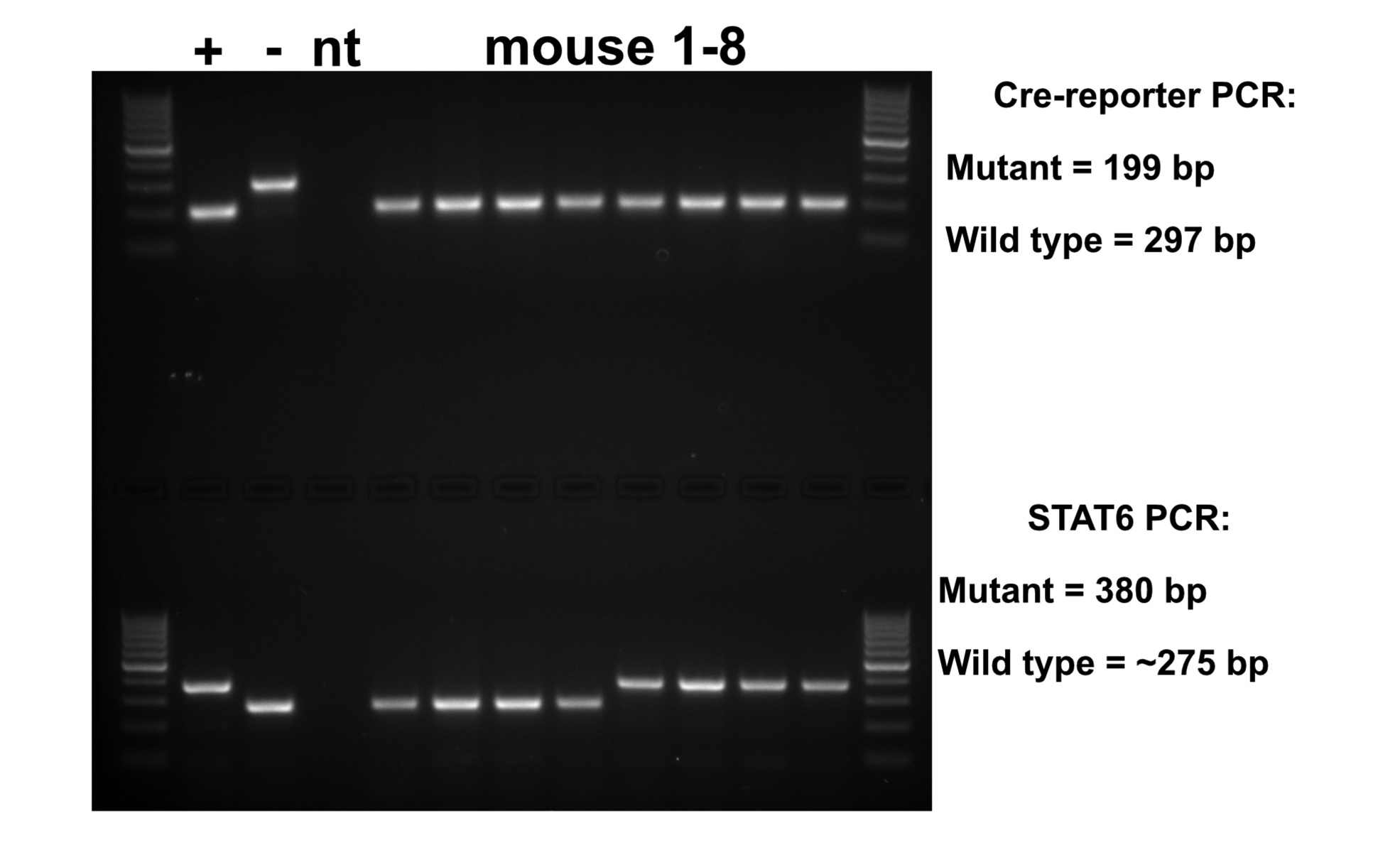

Supplement: S6 Fig — Top, PCR for Cre reporter. Wild-type size = 297bp and mutant (Cre reporter) size = 199bp. Bottom, PCR for STAT6. Wild-type (STAT6) size = 275bp and mutant (STAT6KO) size = 380bp. + = positive control,— = negative control and nt = H2O control. (TIFF) [file ppat.1011347.s006.tiff]

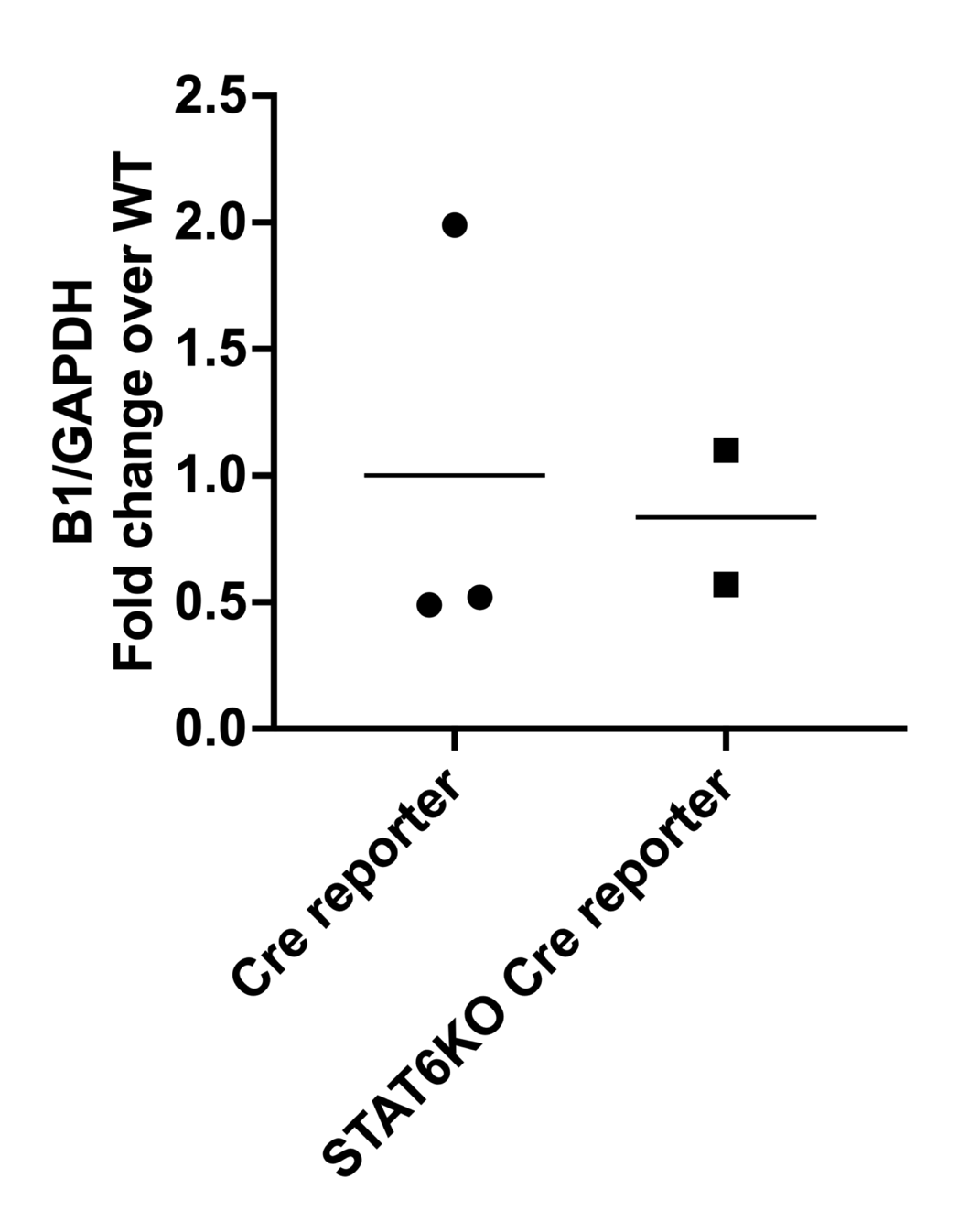

Supplement: S7 Fig — Cre reporter mice or STAT6KO mice were infected intraperitoneally with WTIII parasites. At 3 wpi, brains from these mice were fed to CBA/j mice. At 3 wpi, brains were harvested from CBA/js and CNS parasite burden quantified as in Fig 5B. (TIFF) [file ppat.1011347.s007.tiff]

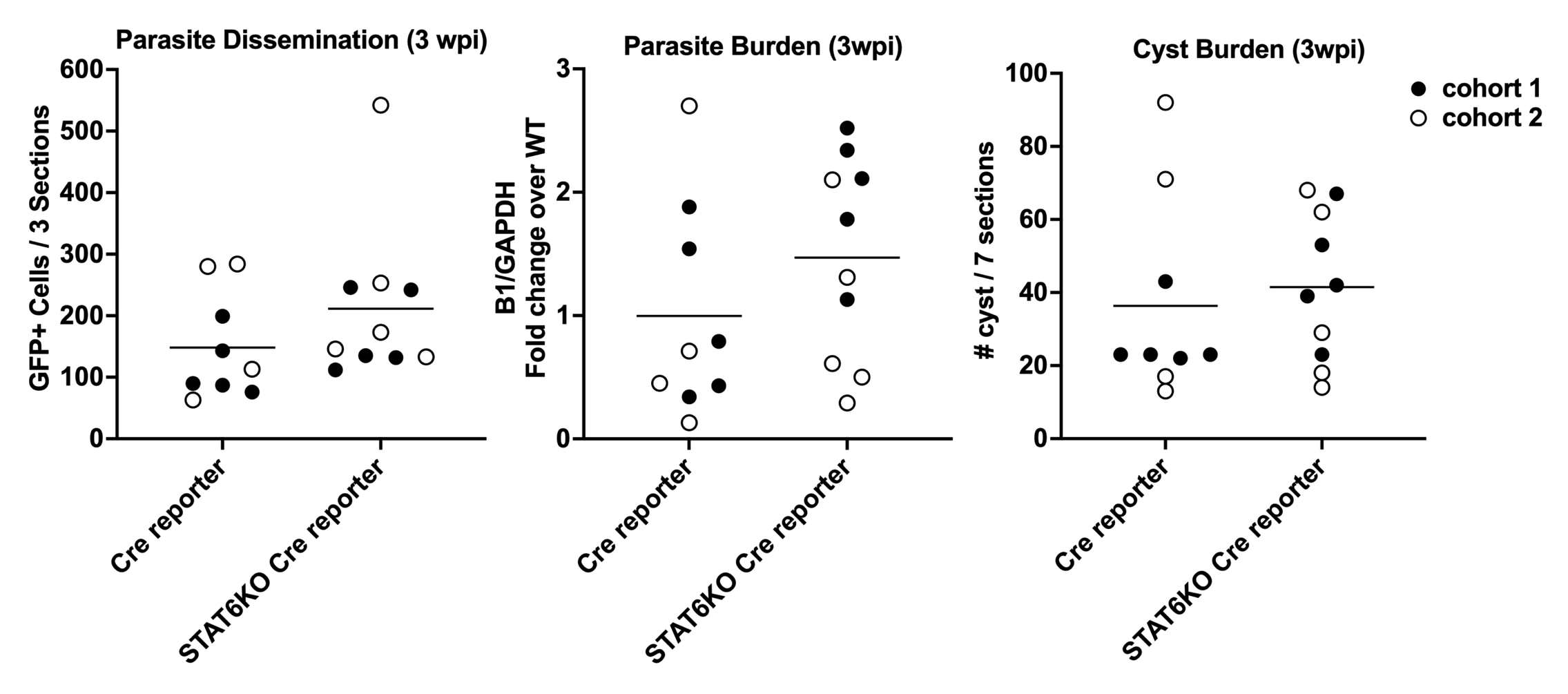

Supplement: S8 Fig — Cre reporter (control) and STAT6KO Cre reporter mice were infected and analyzed at 3 wpi as in Fig 6 except using WTII parasites and staining sections for cyst burden with anti-T. gondii antibody as well. Left, quantification of the total number of GFP+ cells per 3 sections per mouse as in Fig 6A. Middle, quantification of T. gondii gene B1 as in Fig 5B. Right, Quantification of cyst burden (anti-T. gondii antibody+mCherry+DBA+). Bars = mean. Black dots = 1 mouse. N = 4–5 mice per condition per cohort. (TIFF) [file ppat.1011347.s008.tiff]
